# UVA Irradiation Promotes ROS-Mediated Formation of the Common Deletion in Mitochondrial DNA

**DOI:** 10.3390/life16040577

**Published:** 2026-04-01

**Authors:** Gabriele A. Fontana, Navnit K. Singh, Nadezhda Rotankova, Antonia Eichelberg, Michela Di Filippo, Michael R. MacArthur, Susanne Heldmaier, Franziska Wandrey, Hans-Dietmar Beer, Shana J. Sturla, Hailey L. Gahlon

**Affiliations:** 1 Department of Health Sciences and Technology, ETH Zurich, Schmelzbergstrasse 9, 8092 Zurich, Switzerland; fontana.gabriele@gmail.com (G.A.F.); navnitkaur.singh@hest.ethz.ch (N.K.S.); 2 Cellvie AG, Technoparkstrasse 1, 8005 Zurich, Switzerland; 3 Department of Dermatology, University Hospital Zurich, 8952 Schlieren, Switzerland; 4 Mibelle Group, 5033 Buchs, Switzerland; 5 Mibelle Group Biochemistry, 5033 Buchs, Switzerland

**Keywords:** mitochondrial DNA deletion, reactive oxygen species, photoaging

## Abstract

Ultraviolet (UV) radiation from the sun causes adverse skin changes such as premature aging. UV-induced mitochondrial DNA (mtDNA) alterations, including deletions, contribute to photoaging and cellular dysfunction. While the most frequent mtDNA rearrangement is the common deletion (CD), characterized by the loss of nearly one-third of the genome (4977 bp), detailed knowledge of mechanisms governing UV-mediated initiation of the CD and mitigation strategies are lacking. Here, we investigated how increasing UV exposure increases CD levels in human skin fibroblasts via cellular reactive oxygen species (ROS) formation and mtDNA oxidation and demonstrated that antioxidant preconditioning of cells prevents UVA-induced CD accumulation. Conversely, UVB exposure induced cyclobutane pyrimidine dimers (CPDs) without affecting ROS, suggesting an ROS-independent pathway. Using a 3D full-thickness human skin model, we confirmed UVA-dependent CD formation in both the epidermis and dermis. RNA-Seq analysis of UVA-exposed fibroblasts revealed upregulation of mitochondrial DNA replication genes and downregulation of mtDNA repair genes. These findings provide insight into how UVA and UVB differ in detrimental effects on mtDNA, with UVA impacting mtDNA maintenance and transcription via a ROS-dependent mechanism, and provide a physiologically relevant platform to evaluate potential interventions.

## 1. Introduction

Solar radiation exposure can damage skin, causing skin cancer [1,2,3] and premature skin aging [4,5,6]. UVA radiation (320–400 nm) is considered the primary cause of photoaging because it penetrates through the epidermis to the dermis, a connective tissue with collagen-producing fibroblasts required for the skin’s structural integrity [7]. UVB (280–320 nm) has a lower penetration depth, reaching the basal layer of the epidermis, but also has direct and indirect effects on both the dermal and epidermal layers of skin [8]. UVA exposure generates reactive oxygen species (ROS), such as hydrogen peroxide, hydroxyl radicals, and singlet oxygen, and prolonged exposure can overwhelm the body’s antioxidant defenses and induce oxidative stress, leading to cellular damage. UVA forms oxidatively generated lesions, particularly 8-oxo-7,8-dihydro-2′-deoxyguanosine (8-oxo-dG), whereas UVB forms cis-syn-cyclobutane dimers and (6-4) pyrimidine photoproducts [9], making both potent mutagens that damage genomic DNA [10,11]. However, the molecular pathways through which UV-induced ROS contribute to photoaging remain only partially elucidated [12,13].

Mitochondria are organelles with a primary function of generating energy for the cell in the form of ATP via the electron transport chain, making them the site of the highest ROS turnover in cells [14]. In humans, each mitochondrion contains several copies of a circular DNA molecule, named mitochondrial DNA (mtDNA), of 16,569 base pairs, which encodes for 37 genes, including 13 oxidative phosphorylation proteins, 22 tRNAs, 2 rRNAs, and the Humanin micropeptide. Mutations in mtDNA, including point mutations and small- and large-scale deletions, can disrupt mitochondrial physiology and lead to cellular dysfunction [15]. Mitochondrial DNA (mtDNA) exists in a state of heteroplasmy, in which wild-type (non-mutated or non-deleted) and mutated or deleted mtDNA molecules coexist within the same cell [16]. UV-induced mtDNA mutations have been found in photoaged human skin cells [17,18,19], and large-scale deletions of the mitochondrial genome are implicated in UV radiation-induced photoaging of the skin [19,20,21,22,23,24]. Mitochondria have been found to play a critical role in skin aging [17,25,26], and their dysfunction is recognized as one of the hallmarks of aging [27], making them a viable target for anti-aging interventions [28,29].

The common deletion (CD) is the most prevalent large-scale mitochondrial deletion, involving the removal of almost a third (4977 base pairs) of the mitochondrial genome. The mtDNA region deleted by the CD is flanked by two 13-base-pair repeats, with one repeat being retained in the deletion. This deletion was first reported in 1989 in a patient with Kearns-Sayre syndrome (KSS) [30]. UV light appears to promote formation of the mtDNA CD (24), and higher levels of CD were observed to form after UV exposure of skin and are associated with photoaging [22,31,32,33,34]. We previously suggested the CD as a candidate biomarker for cancer-associated fibroblasts [35]. To explore the potential of the CD as a biomarker for UVA-induced photoaging, we require molecular characterization of UV-induced CD formation and evaluation of its ability to respond to possible interventions, such as antioxidants, in the case of ROS-dependent formation.

Several theories have been proposed regarding details of how the CD in mtDNA forms, including the replication-slippage model [30], the copy-choice recombination model [36], and DNA repair-associated deletion formation [37,38]. The replication-slippage model and the copy-choice recombination model depend on replication of mtDNA, which consists of a heavy strand (H-strand) and a light strand (L-strand), which are replicated from their respective origins [39]. The replication-slippage model proposes that fork stalling during H-strand synthesis promotes mis-annealing of single-stranded mtDNA regions. The 13-base pair 3′ repeat of the H-strand mis-anneals with the downstream 5′ repeat, forming a loop that can be extruded from the mtDNA, leading to the formation of the mtDNA CD. Interestingly, a modified replication-slippage model suggests that the singlet oxygen generated, for example by UVA exposure, induces base damage in guanine-rich regions, leading to strand breaks necessary for the degradation of the looped-out DNA (29). The formation of the CD through the copy-choice recombination model involves the synthesis of the mtDNA L-strand. It is proposed that mtDNA deletions can be formed during the repair of double-strand breaks through mechanisms such as homologous recombination or nonhomologous end-joining, with the latter potentially contributing to deletions lacking direct repeats [37,38,40]. Given the overlap between replication and repair pathways in the mitochondria, it is also possible to envision a mechanism that relies on replication-repair crosstalk [15,41]. While these mechanisms could explain how the mtDNA CD is formed, involving oxidative stress [42,43] and mtDNA replication fork stalling [15], data linking how UV radiation initiates the formation of the CD are still lacking.

In this study, we characterized the degree of formation of the mtDNA CD in human skin fibroblasts following UVA or UVB exposures. To probe the role of oxidative stress in the initiation of CD formation, we simultaneously monitored changes in ROS and mtDNA base damage in mtDNA. Furthermore, we supplemented fibroblasts with antioxidants in combination with UVA exposure to evaluate its impact on cellular ROS and how it relates to the formation of the CD. By assessing changes in gene expression in UVA-exposed fibroblasts, we identified clusters of genes that are up- and downregulated in response to UVA irradiation. This study reveals how UVA and UVB differentially induce CD formation and highlights its correlation with oxidative stress. Additionally, it shows that UVA specifically shapes transcriptional responses, providing new insights into the mitochondrial contribution to photoaging.

## 2. Materials and Methods

### 2.1. 2D Fibroblast Cell Culture

BJ-5ta human foreskin fibroblasts (American Type Culture Collection, CRL-4001, Manassas, VA, USA) were maintained in a 4:1 mixture of Dulbecco’s DMEM high glucose (DMEM; Thermo Fisher Scientific, Waltham, MA, USA) and Medium 199 (Thermo Fisher Scientific), and supplemented with 10% fetal bovine serum (FBS; Thermo Fisher Scientific) and 0.01% mg/mL Hygromycin B from Streptomyces hygroscopicus (Sigma-Aldrich, Merck KGaA, Darmstadt, Germany). KSS human skin fibroblasts from a donor with Kearns-Sayre Syndrome were enucleated and fused to 143B osteosarcoma cells and were kindly provided by Professor Ivan Tarassov (University of Strasbourg) (43). KSS fibroblasts were cultured in DMEM (ThermoFischer Scientific) supplemented with 10% FBS (Sigma-Aldrich) and 1% Penicillin-Streptomycin (Thermo Fisher Scientific). Both cell lines were cultured at 37 °C in 5% CO_2_. Cells were routinely passaged every 2–3 days. For passaging, cells were washed once with Dulbecco’s phosphate-buffered saline (DPBS, Thermo Fisher Scientific), then detached with Trypsin-EDTA (0.25%), phenol red (Thermo Fisher Scientific). Trypsin was neutralized with the corresponding medium, and cells were passaged in a new Petri dish (Greiner Bio-One, Kremsmünster, Austria).

### 2.2. Generation of Human Skin Equivalents

Skin equivalents were generated per the protocol described by Berning, Prätzel-Wunder et al. 2015 [44]. In summary, for the dermis-like compartment, human dermal fibroblasts (HDF) were seeded onto 12-well translucent ThinCerts™ (high-density 0.4 μm pores, 11.31 cm^2^ culture area; Greiner Bio-One, Kremsmünster, Austria) at a density of 5 × 10^5^ cells per well on days 1, 3, and 5 and placed in deep-well plates (VWR International, Radnor, PA, USA). The HDF were incubated at 37 °C, 5% CO_2_ and 20% O_2_ over a total culture time of 4 weeks, with a resulting input of HDFs of 1.5 × 10^6^ cells per transwell insert. HDFs were cultured in 3:1 DMEM/Ham’s F12 (FAD) containing 10% FBS (Sigma-Aldrich) and 1% Antibiotic-Antimycotic (100×) (Thermo Fisher Scientific), supplemented with 200 μg/mL 2-phospho-l-ascorbic acid (Sigma-Aldrich) and additional recombinant human proteins: 1 ng/mL transforming growth factor β-1 (TGFβ-1; Thermo Fisher Scientific), 2.5 ng/mL epidermal growth factor (EGF; Thermo Fisher Scientific), 5 ng/mL basic fibroblast growth factor (bFGF; Peprotech, Thermo Fisher Scientific, Cranbury, NJ, USA), and 5 μg/mL insulin (Sigma-Aldrich). The medium was changed every other day for 4 weeks. For the epidermis-like compartment, 2.5 × 10^5^ human primary keratinocytes (HPKs) were seeded per dermal equivalent in FAD medium containing 10% FBS and 1% antibiotics/antimycotics, supplemented with 200 μg/mL 2-phospho-l-ascorbic acid-trisodium salt (Sigma-Aldrich), 0.4 μg/mL hydrocortisone (Sigma-Aldrich), and 1 × 10^−10^ M cholera toxin (Sigma-Aldrich). After 3 days of submersed growth, the epithelial co-cultures were air-lifted, and the medium was changed every other day for 2 weeks.

### 2.3. UVA and UVB Irradiation

**Figure 1:**
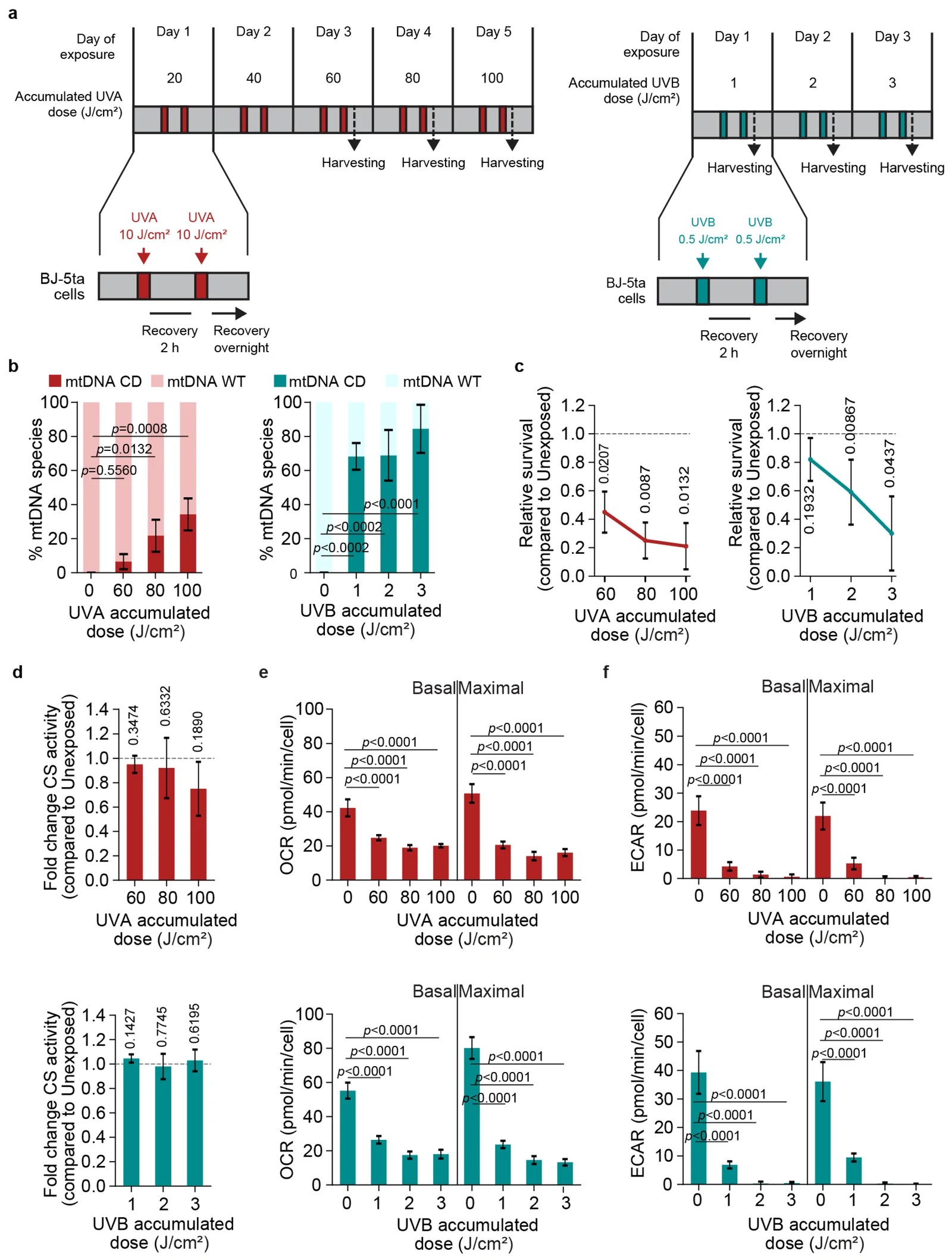
UV light-dependent induction of the mitochondrial DNA common deletion (mtDNA CD) in human fibroblasts. (**a**) Schematic representations of the UVA (left) and UVB (right) exposures performed on the BJ-5ta human fibroblast cell line. Cells in medium devoid of FBS and antibiotics were irradiated with UV twice per day with the indicated doses of UVA (red bars) or UVB (cyan bars). During recovery intervals (grey bars), cells were placed in an incubator with a medium containing FBS and antibiotics. Cells were harvested 30 min after the last indicated irradiation. Dotted vertical arrows indicate harvesting timepoints and horizontal arrows indicate overnight recovery periods. (**b**) Relative quantification of wild-type mtDNA (mtDNA WT, light bars) and mtDNA harboring the common deletion (mtDNA CD, dark bars) using qPCR in Unexposed and UVA- (left) or UVB-exposed (right) cells. (**c**) Relative survival of BJ-5ta cells irradiated with indicated doses of UVA (left) or UVB (right) compared to Unexposed cells (grey dotted line). (**d**) Citrate synthase activity in UVA- (top) and UVB-exposed (bottom) BJ-5ta cells. The citrate synthase activity in UVA and UVB exposed cells is expressed as fold-change per well to Unexposed control cells (grey dotted line). (**e**) Oxygen consumption rates (OCR) at basal and stimulated (maximal) ETC activity in UVA (top) and UVB (bottom) exposed cells. (**f**) Extracellular acidification rates (ECAR) at basal and maximal ETC activity in UVA- (top) and UVB- (bottom) exposed cells. For panels (**b**,**e**,**f**), statistical significance was determined using one-way ANOVA followed by Dunnett’s multiple comparisons test against unexposed control. For panels (**c**,**d**), statistical significance was assessed using one-sample *t*-tests comparing each exposure condition to unexposed control. For (**b**–**d**) *n* = 3 biological replicates were used and for (**e**,**f**) *n* =  7 biological replicates. Error bars represent standard deviation and *p*-values are indicated in the figure.

**Figure 3:**
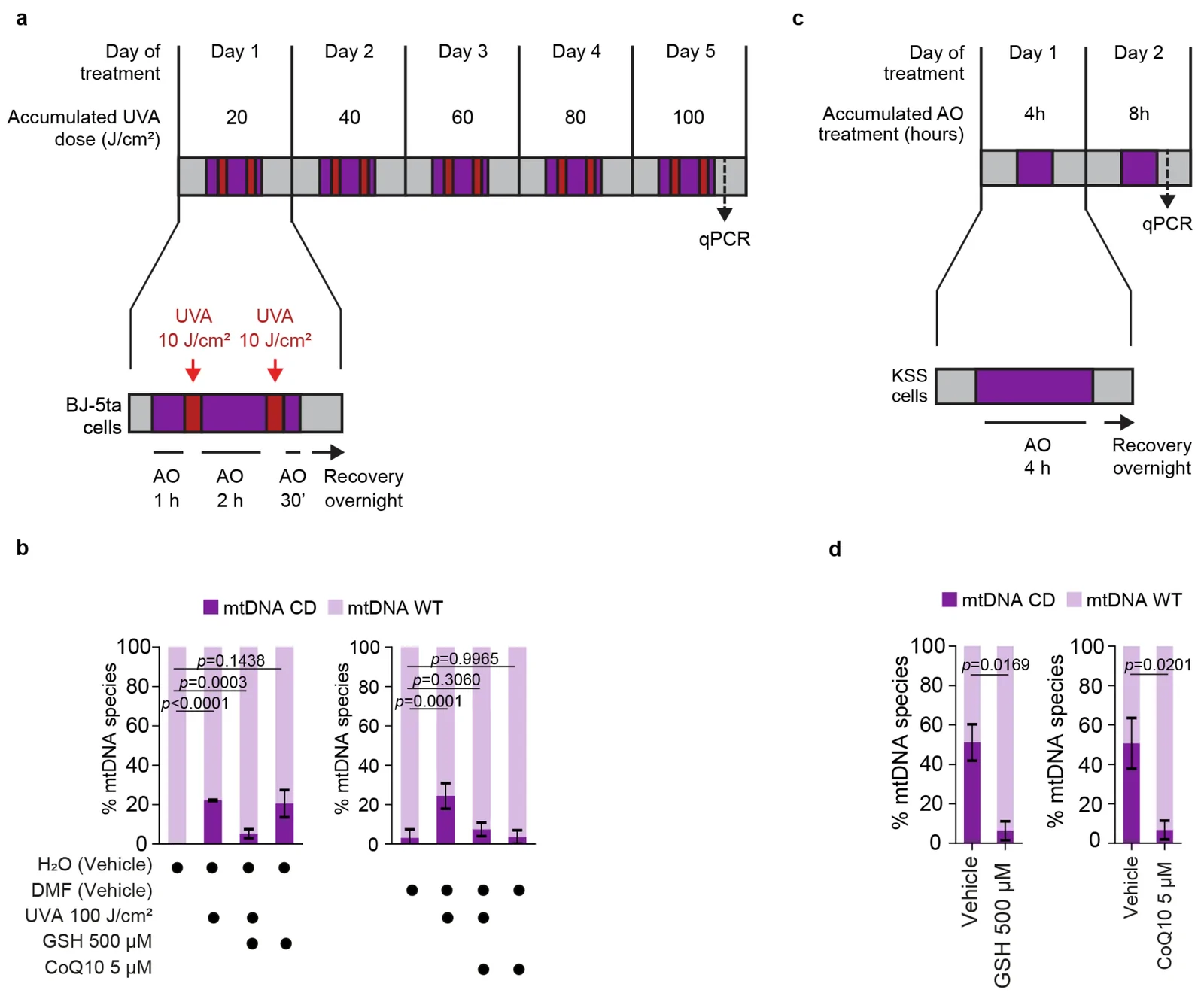
Antioxidants reduce the formation or accumulation of the mtDNA CD upon UVA. (**a**) Schematic representation of the UVA and antioxidant (AO) co-treatments performed on the BJ-5ta human fibroblast cell line. Cells were pre-incubated with AO for 1 h in complete medium (purple bar). The AO-containing medium was removed, and cells were irradiated twice with UVA 10 J/cm^2^ in medium devoid of FBS and antibiotics (red bars). In between irradiations as well as after the last irradiation, cells were incubated with AO for 2 h and 30 min. Harvesting (dotted arrows) was performed 30 min before and after the first last AO incubation, assessing the ROS content at the start (S) or end (E) of the exposure regime. Sampling for qPCR-based assessment of the mtDNA CD content is also indicated. (**b**) Relative quantification of wild-type mtDNA (mtDNA WT, light bars) and mtDNA harboring the common deletion (mtDNA CD, dark bars) by qPCR of BJ-5ta cells exposed to UVA and co-treated with glutathione (GSH, left), Coenzyme Q10 (CoQ10, right) and/or the corresponding vehicles H_2_O or Dimethylformamide (DMF) as in the scheme in Figure 3a. Left panel: *n* = 3; right panel *n* = 2. (**c**) Schematic representation of the AO treatments in KSS cells. Cells were acutely exposed to AO for 4 h per day in complete medium for 2 days. Cells were harvested 30 min after the last incubation, and their ROS and mtDNA CD contents were assessed by qPCR assays. (**d**) Relative quantification of mtDNA WT (light bars) and mtDNA CD (dark bars) by qPCR in KSS cells treated with vehicle or with the indicated AO for 8 h, for 4 h per day. For panel (**b**,**d**) *n* = 3 biological replicates. Significance was determined using one-way ANOVA followed by Dunnett’s multiple comparisons test against unexposed control. Error bars represent standard deviations and *p*-values are indicated in the figure.

**Figure 5:**
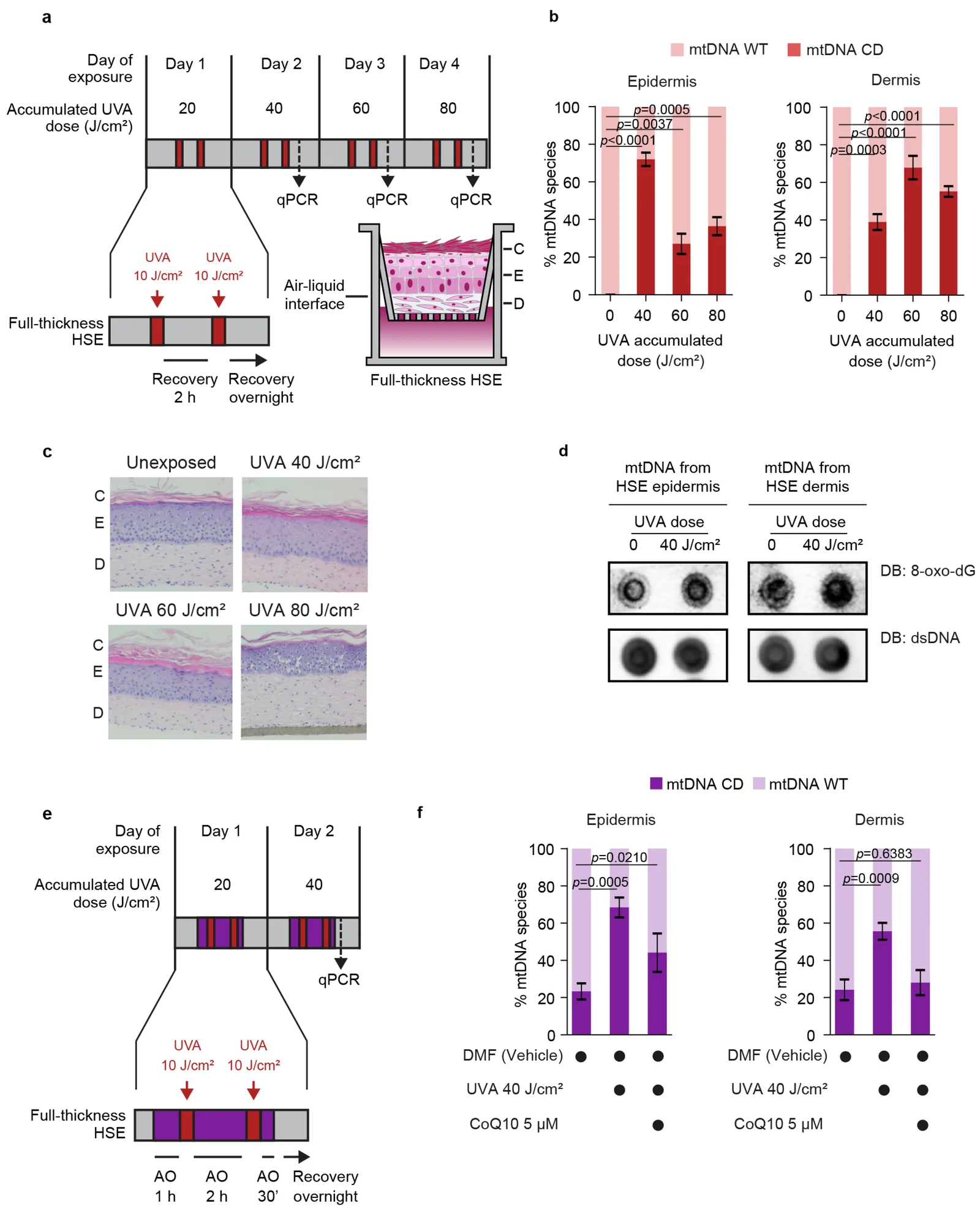
UVA irradiation induces ROS-dependent accumulation of the mtDNA CD in the epidermis and dermis of full-thickness HSE. (**a**) Schematic representations of the UVA exposure regime performed on three-dimensional full-thickness human skin equivalents (HSEs). HSEs were irradiated twice per day with the indicated doses of UVA (red bars), interrupted by recovery intervals (grey bars). Harvesting was performed 30 min after the last indicated irradiation. Dotted vertical arrows indicate harvesting timepoints and horizontal arrows indicate overnight recovery periods Full-thickness HSEs (inlet) are composed of primary human fibroblasts and keratinocytes, constituting the dermis (D) and epidermis (E), respectively, seeded on a plastic air-lifted scaffold. Cellular proliferation and interactions between the two skin layers lead to formation of the corneum layer (C), reconstituting the structure and functionality of human skin. (**b**) Relative quantification of wild-type mtDNA (mtDNA WT, light bars) and mtDNA harboring the common deletion (mtDNA CD, dark bars) by qPCR in HSEs exposed to increasing doses of UVA. The dermis (left) and epidermis (left) were analyzed separately. (**c**) Representative microscopy images of hematoxylin-eosin-stained HSE sections, in unexposed conditions or exposed to the indicated doses of UVA. (**d**) Dot blots performed with mtDNA extracted from the epidermis and dermis of HSEs, untreated or treated with 100 J/cm^2^ UVA and hybridized with anti-8oxodG and anti-dsDNA antibodies. (**e**) Schematic representation of the UVA and CoQ10 co-treatments performed on HSE. Cells were pre-incubated for 1 h with 5 µM CoQ10 in complete medium (purple bar). The CoQ10-containing medium was removed, and HSEs were irradiated twice with UVA 10 J/cm^2^. In between irradiation as well as after the last irradiation, cells were incubated with CoQ10 for 30 min. Harvesting (dotted arrows) was performed 30 min after removing the CoQ10-containing medium. (**f**) Relative quantification of mtDNA WT (light bars) and mtDNA CD (dark bars) by qPCR of HSEs exposed to UVA and co-treated 5 µM CoQ10 and/or the corresponding vehicle scheme specified in Figure 5f. The dermis (right) and epidermis (left) were analyzed separately. *n* = 3 biological replicates in panel (**b**,**f**). Significance was determined using one-way ANOVA followed by Dunnett’s multiple comparisons test against unexposed control. Error bars represent standard deviations and *p*-values are indicated in the figure.

Cells were grown to 70–80% confluency in 15 cm Petri dishes. Prior to irradiation, the culturing medium was changed to serum-free DMEM (Thermo Fischer Scientific) without antibiotics. Cells were irradiated in a UV irradiation chamber (BS-02 Opsytec Dr. Grödel GmbH, Ettlingen, Germany) according to the schemes shown in the corresponding figures (Figure 1a, Figure 3a and Figure 5a,e). For UVA, dishes were covered with a soda-lime glass (150 × 25 mm, Faust Laborbedarf AG, Schaffhausen, Switzerland) to filter the remaining minor fraction of UVB emitted by the irradiation system. A dosimeter was placed internally to the irradiation chamber and was used to monitor the UVA dose. For UVB exposure, irradiation for 3 min 20 s with four 5 mV UVB lamps (Opsytec Dr. Grödel GmBH) resulted in an irradiation exposure level of 0.5 J/cm^2^. UVB exposures were performed without a cover on the dishes. For experiments involving antioxidants, reduced L-glutathione (Sigma-Aldrich) dissolved in H_2_O (final concentration 50 µM) or coenzyme Q10 (CoQ10, Sigma-Aldrich) in dimethylformamide (DMF; Sigma-Aldrich, final concentration 500 µM) were added to the serum-free medium. The serum-free medium was replaced with culturing medium after irradiation. During irradiation, all control dishes were kept at room temperature within a laminar flow hood.

### 2.4. Human Skin Equivalent Staining

Skin equivalent samples were fixed overnight in formalin 4%, dehydrated automatically and embedded in paraffin (Leica EG 1150; Leica microsystems, Wetzlar, Germany). Paraffin blocks were cut into 5 μm thick sections and stained with haematoxylin and eosin (H&E) using the HistoCore SPECTRA ST (Leica Microsystems) according to its standard protocols or analyzed by immunohistochemistry or immunofluorescence.

### 2.5. Immunohistochemistry

For specific staining of paraffin sections, tissue sections were deparaffinized in xylene and rehydrated by incubating in decreasing concentrations of ethanol (100%, 96%, 80%, 70% and 50%) for 2 min, followed by water for 2 min. The activity of endogenous peroxidase was blocked by 10 min of incubation in 3% peroxidase-blocking solution (hydrogen peroxide 30% in H_2_O; Merck KGaA, Darmstadt, Germany). For antigen retrieval, slides were heated in a steamer for 30 min in antigen retrieval solution, sodium citrate buffer, pH 6.0 (10 mmol/L sodium citrate, 0.05% Tween20). Sections were then blocked for 1 h at room temperature with 5% bovine serum albumin in PBST (0.05% Tween 20 in PBS, blocking solution) and incubated overnight at 4 °C with the primary antibody (Ki67; Abcam, cat. no. ab833, rabbit, 1:100, Cambridge, UK) diluted in the blocking solution. Biotinylated secondary antibody (Anti-Rabbit IgG BIOT, cat. no. 4010-08; SouthernBiotech, Birmingham, AL, USA) was applied for 1 h at room temperature. Staining was visualized with the AEC substrate (Dako, Agilent Technologies, Glostrup, Denmark); nuclei were stained using Mayer’s hematoxylin (Kantonsapotheke, Zürich, Switzerland). Slides were mounted with Faramount aqueous mounting medium (Dako) and scanned using an Aperio ScanScope (Leica Biosystems) or with the PhenoImager Vectra Polaris (Akoya Biosciences, Marlborough, MA, USA).

### 2.6. mtDNA and RNA Extraction

Cells were harvested by scraping and pelleted by centrifugation (3 min, 4000 rcf, at room temperature). Cell pellets were either processed immediately or stored at −20 °C. RNA was isolated from cell pellets using the RNeasy Mini Kit (Qiagen, Hilden, Germany) following the manufacturer’s instructions. The RNA preparation included an on-column RNase-Free DNase (Qiagen) treatment prior to elution. mtDNA was isolated from cell pellets as previously described [45]. Concentrations of purified RNA and mtDNA were assessed using a NanoDrop (Thermo Fisher Scientific) spectrophotometer. For mtDNA extraction from HSE, epidermal and dermal layers were separated, and cells were lysed in Buffer P1 supplemented with RNAse (Qiagen) using a TissueLyser (Qiagen) for 3 min at 30 Hz. The lysates were then processed similarly to the cell pellets, as described above.

### 2.7. Quantitative Real-Time PCR (qPCR) for Quantitation of the CD

Purified and RNA-free mtDNA was quantified for the CD by qPCR as described previously [35,45]. In brief, for each sample 5–10 ng mtDNA, 0.4 μM forward primer, 0.4 μM reverse primer, 6 μL GoTaq 2× qPCR Master Mix (Promega, Madison, WI, USA), and nuclease-free water were prepared to a final volume of 12 μL per sample. Three technical replicates were analyzed for each mtDNA and primer combination. The CD primers bind 5 kb apart on wild-type mtDNA and do not amplify under the qPCR conditions used; a 200 bp product is obtained exclusively from deleted molecules where the intervening 4977 bp are absent. The CD primers used in this study were adopted from Phillips et al. [41], who validated specificity by agarose gel electrophoresis, confirming amplification of a single band of expected size (Phillips et al.). In our qPCR assay, melt curve analysis confirmed a single specific amplification product, consistent with these findings. Random DNA fragmentation from apoptotic cells is unlikely to generate a false-positive signal, as this would require breaks at precisely both 13 bp repeat positions flanking the deletion, effectively recapitulating the deletion junction itself. All primers used in this study are were adapted from [35,41]. And arelisted in Table S1 and were obtained from Eurogentec (Eurogentec, Seraing, Belgium). All qPCRs were conducted on the Rotor-Gene 6000 (Corbett Research, now Qiagen, Sydney, Australia) device, following a standard qPCR protocol. From the qPCR data, we derived values corresponding to deleted mtDNA and undeleted mtDNA for each sample. We accounted for their relative change from the total mtDNA in each sample by subtracting the Ct value for total DNA from the Ct values for each deleted or undeleted DNA. We then expressed changes in each of these values, i.e., deleted or undeleted mtDNA for each sample, as proportional fold changes in the corresponding values in untreated cells. A detailed protocol including the experimental method, derivation of the relative values, and their statistical treatment is published in [45]. Finally, Genomic DNA contamination was routinely assessed by qPCR on a genomic region of the ACTB gene, which resulted in low or no signal.

### 2.8. Quantitative Real-Time PCR (qPCR) for the Analysis of Gene Expression Levels

RNA expression was quantified by qPCR as described previously [35]. The High-Capacity cDNA Reverse Transcription Kit (Thermo Fisher Scientific) was used to reverse transcribe 1–2 μg purified RNA into cDNA according to the manufacturer’s instructions. For each sample of 4–6 ng cDNA, 6 µL HOT FIREPol EvaGreen qPCR Mix (Solis Biodyne, Tartu, Estonia), 0.4 μM forward primer, 0.4 μM reverse primer, and nuclease-free water were prepared to a total volume of 12 μL. All primers used in this study are listed in Table S1 and were obtained from Eurogentec, Belgium. All qPCRs were conducted on the Rotor-Gene 6000 (Corbett Research) device, using the following protocol for each cycle: 95 °C for 30 s, 60 °C for 1 min, 40–50 cycles, with fluorescence acquisition at the end of the last amplification step. Expression levels for each biological replicate were assessed using the delta-delta Ct method. For nuclear genome-encoded target genes, amplification of GAPDH or ACTB housekeeping genes were used as normalizers. For mtDNA-encoded target genes, the amplification of an untranslated region of the polycistronic mtDNA-encoded RNAs was used as a normalizer.

### 2.9. RNA-Sequencing

RNA sequencing was performed by the Functional Genomics Center Zürich. RNA was extracted using the Qiagen RNeasy Mini Kit following the manufacturer’s protocol. Extracted RNA was prepared for sequencing using the Illumina TruSeq Stranded mRNA Library Prep assay following the manufacturer’s protocol. Sequencing was performed on the Illumina NovaSeq 6000 (Illumina, San Diego, CA, USA) using the S1 Reagent Kit v1.5 (100 cycles) as per the manufacturer’s protocol. Demultiplexing was performed using Illumina bcl2fastq Conversion Software (version 2.20.0.422). Individual library size ranged from 20 million to 26 million reads.

### 2.10. Measurement of Reactive Oxygen Species (ROS)

A total of 1.5 × 10^4^ cells were seeded in 96-well-plates and assayed at a confluency of 70–80%. Culturing medium was removed from each well and replaced with 200 μL of 50 μM 2′,7′-Dichlorofluorescin diacetate (DCF, Sigma-Aldrich) solution in PBS (1×). Plates were incubated for 30 min (37 °C, 5% CO_2_), the solution was removed, and 30 μL Trypsin (0.25%), phenol red (Thermo Fisher Scientific) was added. Following 3–5 min incubation for detachment of the cells, 120 μL PBS (1×) was added, and fluorescence was acquired using a CytoFLEX flow cytometer (Beckman Coulter, Brea, CA, USA). Data were normalized internally by gating. To determine the ROS fold-change, the intensity of the cell-free blank control was first subtracted from each condition. Fold-change was determined from the ratio of intracellular ROS levels in UV-irradiated versus unexposed cells. Each condition was assayed in three biological replicates, and each sample was tested in three technical replicates.

### 2.11. Citrate Synthase Assay

A total of 1.5 × 10^4^ cells were seeded in 96 well-plates and assayed at a confluency of 70–80%. The activity of the citrate synthase enzyme, a proxy for cellular mitochondria content, was measured using the Citrate Synthase Activity Assay Kit (Abcam) following the manufacturer’s instructions. The colorimetric readings were acquired using the Infinite 200 PRO microplate reader(Tecan, Männedorf, Switzerland) and normalized to a cell-free blank and to the amounts of harvested cells for each sample. Each condition was assayed in three biological replicates, and each sample was tested in three technical replicates.

### 2.12. Seahorse Assay

Basal and UV-induced changes in oxygen consumption rate (OCR) and extracellular acidification rate (ECAR) were measured using a Seahorse XF96 Extracellular Flux Analyzer (Agilent Technologies, Santa Clara, CA, USA). Assays were performed in Seahorse XF Assay Medium (pH 7.4) supplemented with glucose (10 mM), sodium pyruvate (1 mM) and L-glutamine (2 mM). Following each injection, four measurement cycles were performed, each consisting of 3 min of mixing, 2 min of waiting, and 3 min of measurement.

A basal measurement was performed followed by oligomycin (1 μM), 4-(trifluoromethoxy)phenylhydrazone (FCCP, 1.5 μM) and rotenone/antimycin A (2 μM) injections. All OCR and ECAR values were normalized to cell count as measured by nuclear staining with Hoechst 33342 (Thermo Fisher Scientific), followed by automated cell counting using a Cytation 5 Cell Imaging Reader (BioTek, Agilent Technologies, Winooski, VT, USA) and Gen5 software (BioTek).

### 2.13. Dot Blot

Dot blots were performed on mtDNA extracted from UV-irradiated and unexposed cells as a qualitative indication of the presence of 8-oxo-dG and CPD in the mtDNA. All reagents used in this procedure, from the extraction to the solutions used until the incubation with the primary antibody, were supplemented with 350 μM 8-hydroxyquinoline (Sigma, stock dissolved in 1:1 EtOH; H_2_O), a chelating antioxidant [46] used to prevent artifactual formation of oxidized mtDNA bases. Unless stated otherwise, all steps of this procedure were performed at room temperature. A total of 2 μL of isolated mtDNA, corresponding to 20 ng of mtDNA, was dotted on pre-cut nitrocellulose membranes (Thermo Fisher Scientific) and dried for 2 h at 60 °C. The samples were then immerged in 5 mL of blocking buffer, composed of 0.5% BSA (Thermo Fisher Scientific) in TBS-T (20 mM tris-HCl, 150 mM NaCl pH 7.5, 0.05% Tween20) (Thermo Fisher Scientific), and incubated for 1 h on a shaker. The blocking buffer was then removed, and the membranes were covered in a solution containing the respective primary antibodies (anti-ds DNA (ab27156, Abcam) anti-CPD (Clone TDM-2, Cosmo Bio USA, Carlsbad, CA, USA) and anti-8-OHdG Antibody (15A3) (cat. no. sc-66036; Santa Cruz Biotechnology, Dallas, TX, USA). The samples were then kept overnight on a shaker at 4 °C. From this point on, the solutions used were no longer supplemented with 8-hydroxyquinoline. The membranes were washed 3 times with TBS-T for 10 min and then incubated for 1 h with the secondary antibody (Abcam ab6789, anti-mouse HRP-conjugated raised in goat, 1:2000) in TBS-T containing 0.5% BSA. Then, the membrane was placed on a plastic foil and covered with mixed ECL reagent (Promega) for 5 min. HRP activity was then recorded using a ChemiDoc Imaging System (Bio-Rad, Hercules, CA, USA). Uncropped membrane scans are provided in the corresponding supplementary figures.

### 2.14. Data Analysis

All values are expressed as mean ± standard deviation (SD). Information on the number of biological replicates is given in the figure legends. Differences in mean values between groups were analyzed using GraphPad Prism 10 (GraphPad Software, Boston, MA, USA), and the statistical analyses performed are indicated in the figure legends. In the case of significant differences, the *p* values obtained are shown in the panels, with *p* < 0.05 considered statistically significant.

RNA sequencing analysis was performed using the SUSHI framework [47], which encompassed the following steps: read quality was inspected using FastQC, and sequencing adaptors were removed using fastp; alignment of the RNA-Seq reads using the STAR aligner [48] and with the GENCODE human genome build GRCh38.p13 (Release 32) as the reference [49]; the counting of gene-level expression values using the ‘featureCounts’ function of the R package Rsubread [50]; differential expression using the generalized linear model as implemented by the EdgeR Bioconductor R package, and; Gene Ontology (GO) term pathway analysis using both the hypergeometric over-representation test via the ‘enricher’ function and gene-set enrichment analysis via the ‘GSEA’ function, of the clusterProfiler Bioconductor R package [51]. All R functions were executed on R version 4.0.

## 3. Results

### 3.1. UVA and UVB Induce CD Formation in Human Skin Fibroblasts

First, we evaluated how CD levels changed in response to UVA vs. UVB exposure in cultured human skin fibroblasts. Using a qPCR assay that we previously developed, we simultaneously measured CD, wild-type (WT), and total mtDNA [45]. Exposure regimes (Figure 1a) consisting of sequential short exposures (22 min for UVA and 3 min 20 s for UVB) and longer recovery periods (2 h and overnight recovery periods as indicated in Figure 1a,b), with maximum irradiation of 100 J/cm2 UVA and 3 J/cm^2^ UVB, which were reached on day 5 for UVA or day 3 for UVB. For both UVA and UVB exposures, we observed a dose-dependent increase in the CD (Figure 1b), with UVA-induced CD levels ranging from 6 to 35% at 60–100 J/cm^2^ (Figure 1b, left). As a control, we measured the UV responsive genes MMP1 and COL1A1 and confirmed their expected UVA-induced up- and downregulation [52] by RT-qPCR analysis (Figure S1). We found that UVB induced higher CD levels (70–80% for 1–3 J/cm^2^) than UVA, suggesting differences in initiation processes or kinetics (Figure 1b, right).

To identify a dose of UVA and UVB with comparable effects on cell viability, we monitored cell survival following UVA or UVB exposure (Figure 1c). UVA caused a gradual decrease in cell survival at 60, 80 and 100 J/cm^2^ (Figure 1c, left), whereas UVB induced a more rapid decline between day 1 and 3 of UV irradiation (Figure 1c, right). Nonetheless, the maximum irradiation doses of 100 J/cm^2^ UVA and 3 J/cm^2^ UVB similarly impacted cell viability, with mean cell survival of 30%. To test whether UVA- and UVB-induced mtDNA CD is accompanied by mitochondrial dysfunction, we assessed several parameters. First, we monitored cellular mitochondrial content on the basis of citrate synthase activity [53], which was unchanged by UVA or UVB exposure (Figure 1d). To assess mitochondrial function and potential changes in electron transport chain (ETC) activity, we monitored mitochondrial respiration (ORC) and glycolysis (ECAR) (seahorse assay) [54]. Maximal respiratory conditions were induced with FCCP, a protonophore that collapses the inner mitochondrial membrane gradient (Figure 1e,f). With FCCP, we observed that both UVA (60–100 J/cm^2^) and UVB (1–3 J/cm^2^) reduced OCR compared to unexposed controls, indicating impaired mitochondrial respiration (Figure 1e). ECAR was also reduced after UVA and UVB exposure, indicating decreased glycolytic activity (Figure 1f). Overall, both UVA and UVB disrupted mitochondrial function without altering mitochondrial content, increased mtDNA CD levels, and reduced cellular fitness.

### 3.2. UVA Promotes mtDNA Base Oxidation and the CD

**Figure 2:**
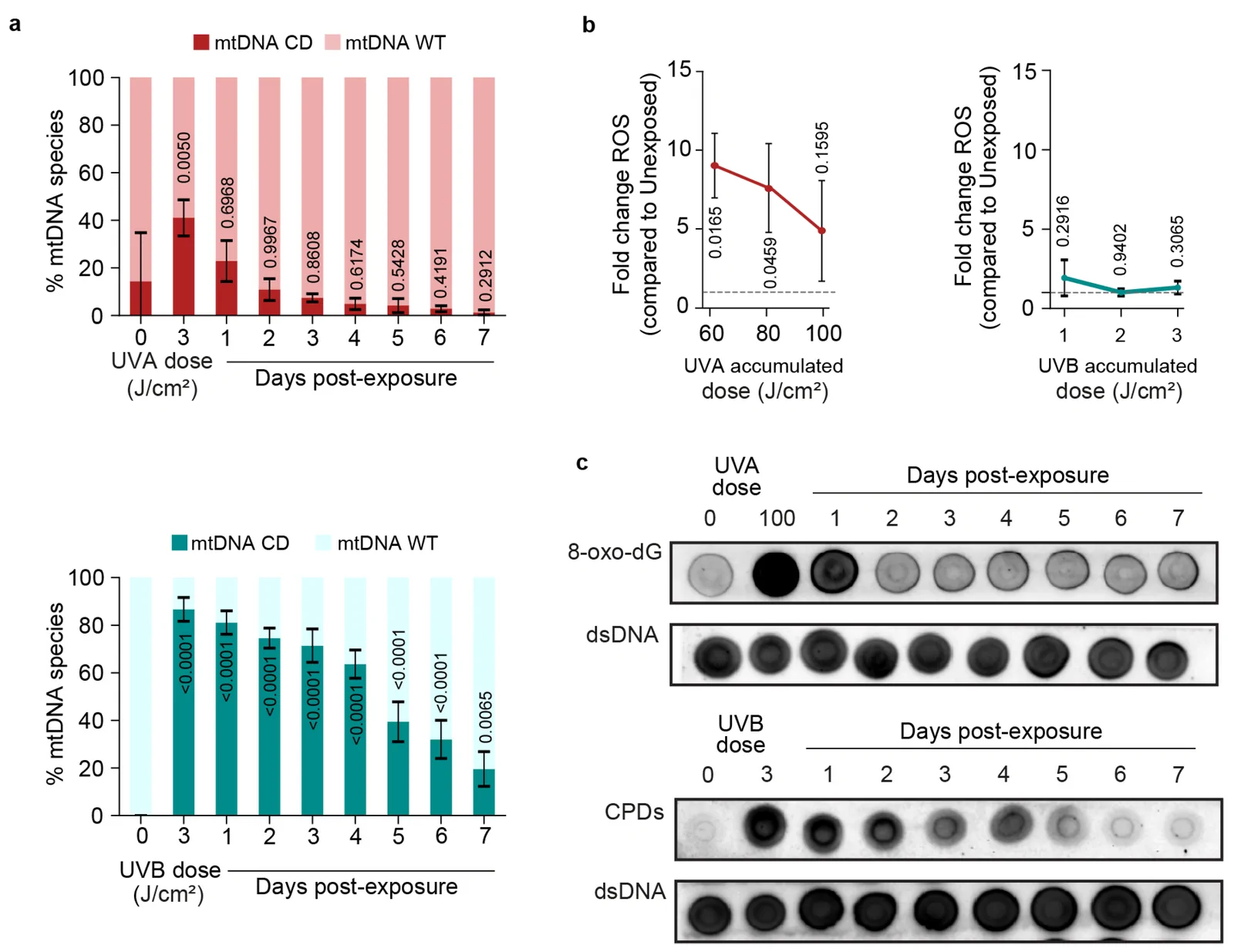
UV radiation induces distinct patterns of mtDNA base damage, leading to diverse kinetic profiles for the recovery of the mtDNA WT pool in the absence of further irradiation. (**a**) BJ-5ta cells were exposed to the indicated doses of UVA (top) and UVB (bottom) according to the scheme in Figure 1a. mtDNA samples were collected every 24 h for seven days after exposure in the absence of further stimuli. Relative quantification of wild-type mtDNA (mtDNA WT, dark bars) and mtDNA harboring the common deletion (mtDNA CD, light bars) by qPCR in unexposed and UVA- (top) or UVB-exposed (bottom) cells. (**b**) Fold-change of cellular ROS content in BJ-5ta cells quantified by measuring the fluorescent 2′, 7′ –dichlorofluorescein (DCF) in UVA- (left) and UVB-exposed (right) cells to unexposed cells (grey dotted line). *n* = 3 biological replicates analyzed by one-sample *t*-tests, comparing each exposure condition to unexposed control. (**c**) Representative dot blots. Dot blots were performed on mtDNA extracted from BJ-5ta cells irradiated with the indicated doses of UVA (top) or UVB (bottom) and collected every 24 h for seven days after exposure in the absence of further stimuli. Hybridization was performed with an anti-8-oxo-dG, an anti-cyclobutane pyrimidine dimer (CPDs), and an anti-double stranded DNA (dsDNA) antibody as a loading control. For (**a**–**c**), *n* = 3 biological replicates. Error bars represent standard deviations. For panels (**a**,**b**), statistical significance was determined using one-way ANOVA followed by Dunnett’s multiple comparisons test against unexposed control and *p*-values are indicated in the figure.

To characterize how UV-induced CD persists after irradiation, we monitored CD levels for seven days following UVA or UVB exposure. For UVA, an accumulated dose of 100 J/cm^2^ resulted in 40% CD, which progressively decreased after irradiation, from 22% on day 1 post-exposure to 1% by day 7 (Figure 2a, top). In contrast, for UVB, 3 J/cm^2^ generated 85% CD (Figure 1b), and these levels declined more slowly, reaching 20% on day 7 post-exposure (Figure 2a, bottom). To determine the origin for these kinetic differences, we measured mtDNA base oxidation and photoproduct formation with a dot blot assay using an anti-8-oxo-dG and an anti-CPD specific antibody, respectively. UVA exposure (100 J/cm^2^) caused a substantial increase in 8-oxo-dG but no detectable CPDs (Figure S2a). We also tracked how cellular ROS and other radical levels changed (H2DCFDA (DCF) assay [55], Figure 2b). We observed a pronounced UVA exposure-induced increase in ROS, with a peak 10-fold rise at 60 J/cm^2^ and lower but pronounced rise at 100 J/cm^2^ (Figure 2b). The observed decrease in DCF fluorescence at the highest cumulative UVA doses may reflect the concurrent reduction in cell viability (Figure 1c) as well as an adaptive antioxidant response in surviving cells. In contrast, UVB did not measurably increase ROS or 8-oxo-dG but induced a strong accumulation of CPDs (Figure S2b).

Based on the UVA/oxidative mtDNA base damage and UVB/photoproduct relationships, we further tested how the post-exposure kinetic profiles of these DNA damage products track with CD formation (Figure 2a). Thus, following UVA exposure, 8-oxo-dG levels returned to the level of the unexposed control within two days (Figure 2c, upper panel, Figure S3a), consistent with the rapid decline of the CD levels. In UVB-irradiated cells, CPD levels persisted longer and returned to the levels of the unexposed control by day 6, matching the slower decline of the CD levels (Figure 2c, lower panel, Figure S3b).

Finally, since oxidative lesions may stall mtDNA replication and potentially initiate CD formation, we performed an in vitro primer-extension assay and observed polymerase γ pausing at the lesion site (Figure S4), consistent with previous reports [56]. Overall, we observed that UVA induced an increase in cellular ROS levels and an accumulation of 8-oxo-dG in normal fibroblasts.

### 3.3. Antioxidants Protect Against CD Formation

Based on our evidence that UVA exposure increases ROS, mtDNA base oxidation and CD accumulation, we tested the capacity of the antioxidants CoQ10 and GSH to reduce UVA-promoted formation of the CD.

Using the DCF assay, we first established that both antioxidants have a similar ability to counteract UVA-promoted ROS (Figure S5a), then we measured CD levels by qPCR for the same conditions (Figure 3a). UVA exposure at 100 J/cm^2^ in the presence of the vehicle increased CD levels to 22% (Figure 3b, left), surprisingly also in the presence of GSH without UVA exposure. UVA-promoted CD formation was reduced to 5% by the addition of GSH before and after UVA irradiation compared to UVA exposure and no GSH addition. Similarly, for the CoQ10 antioxidant, the addition of the antioxidant led to a reduction in the CD level from 22% (UVA-exposed without CoQ10) to 5% with the addition of CoQ10 (Figure 3c, right). These data illustrate that UVA-promoted formation of the CD can be counteracted with the addition of antioxidants in human fibroblasts, linking its formation to ROS.

To further examine the relationship between CD formation and ROS, we tested whether our previous observations were consistent in a more physiologically relevant model of human pathology. The KSS cell line is derived from a skin biopsy from a KSS patient and maintains high CD levels [57]. Furthermore, we established that basal ROS levels in these KSS cells were approximately 2-fold higher than in the BJ-5ta normal fibroblasts. The CD level in this model remains stable at 50% heteroplasmy in basal conditions; however, treatment with the antioxidants GSH (500 µM) or CoQ10 (5 µM) during a 2-day transient exposure (Figure 3c) led to a substantial reduction in CD within 8 h (Figure 3d). This effect coincided with a robust decrease in ROS following antioxidant treatment (Figure S5b). Overall, these data demonstrate an association between oxidative stress, whether UVA-induced (see Figure 3a–c) or endogenous (see Figure 3c,d), and increased CD levels. The data also show that antioxidant treatment is accompanied by a reduction in CD levels.

### 3.4. UVA Exposure Increases the Expression of mtDNA Replication Genes and Concomitantly Decreases the Levels of mtDNA-Encoded Genes

**Figure 4:**
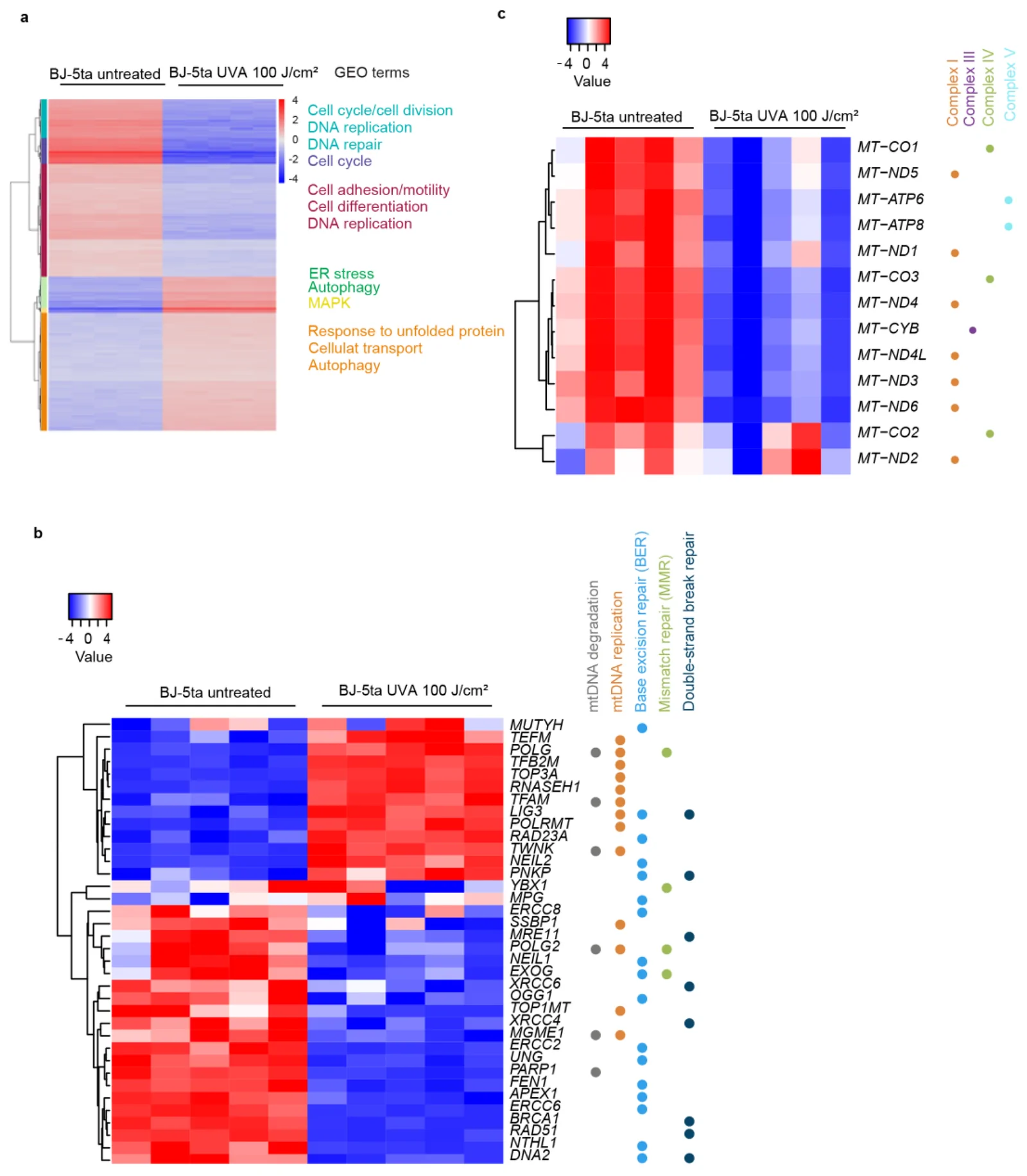
UV-irradiation induces gene expression changes in nuclear DNA-encoded mtDNA maintenance genes and in mtDNA-encoded genes. (**a**) RNA-seq results depicting the genes most affected in their expression upon exposure of BJ-5ta cells with UVA 100 J/cm^2^ according to the scheme in Figure 1a. The 2000 most up- and downregulated genes were binned in 6 clusters (colored bars, left), and GEO terms were assigned to each cluster (right). (**b**) RNA-seq analysis of genes involved in mtDNA maintenance (mtDNA replication, repair and degradation) in unexposed and UVA-exposed BJ5-ta fibroblasts. (**c**). RNA-seq analysis of mtDNA-encoded genes in five unexposed and UVA-treated biological replicates.

Having established that UVA induces CD through ROS production and mtDNA base oxidation, we characterized changes in gene expression. Thus, we performed transcriptomic analysis of nuclear and mitochondrial genes in UVA-exposed fibroblasts on bulk RNA from five biological replicates of each condition. A stringent analysis of global gene expression changes evident from RNA-seq analysis revealed that among the 21,505 genes monitored, 6372 genes were differentially expressed due to the UVA exposure (*p* < 0.01 k correlation of log ratio > 0.5 between biological replicates). Gene ontology analysis on the 2000 most up- and downregulated genes (Figure 4a) led to the identification of six gene clusters: three with upregulated genes, and three with downregulated genes. Interestingly, DNA damage and repair and modulation of the cell cycle were the most deregulated pathways. We then evaluated genes encoding known effectors involved in mtDNA maintenance, and in mtDNA replication, repair and degradation, revealing that genes involved in mtDNA replication are generally upregulated by UVA, while genes involved in mtDNA repair are mostly downregulated (Figure 4b). By monitoring genes relevant for the corresponding pathways in the nuclear genome, we found a similar downregulation for genes encoding for DNA repair (Figure S6a). However, genes encoding enzymes involved in nuclear DNA replication exhibited an opposite trend compared to those on the mitochondrial genome, which were also downregulated by UVA (Figure S6b). Furthermore, the qPCR analysis of selected genes involved in mtDNA degradation, replication and repair showed consistent results, suggesting that genes involved in mtDNA replication and degradation were generally upregulated upon UVA irradiation, whereas most repair-related genes were downregulated or unchanged (Figure S6c).

For all 13 protein-coding mtDNA genes, UVA exposure generally resulted in downregulation (Figure 4c and Figure S6d). Finally, we evaluated gene expression changes in response to UVB irradiation (3 J/cm^2^) for the same genes shown in Figure S6c,d. While we observed similar deregulation in mtDNA-encoded genes, the expression changes in nuclear-encoded genes involved in mtDNA maintenance were specific to the type of UV exposure (Figure S7a,b). Overall, we observed that UVA exposure led to the upregulation of genes involved in mtDNA degradation, the downregulation of repair-related genes, and a reduction in mtDNA-encoded gene expression.

### 3.5. UVA Induces CD Formation in Keratinocytes and Fibroblasts in a 3D Human Skin Equivalent Model

Having established in a 2D cell line that UVA promotes ROS generation and increases CD formation, we further tested their physiological relevance with a 3D tissue-engineered, organotypic full-thickness human skin equivalent (HSE) model. To construct the HSE, human primary keratinocytes were cultured on a natural dermis-like compartment. The dermis-like compartment is derived from triple seeding of human primary fibroblasts and their synthesis of an extracellular matrix within four weeks. To induce keratinocytes, the HSEs were exposed to the air–liquid interface after three days. This model results in epidermal and dermal layers that are histologically similar to native human skin. Since this model is constructed using a fibroblast-derived extracellular matrix instead of plastic, it provides a more physiologically relevant microenvironment for the skin cells [44]. The UVA exposure regime of the HSEs involved sequential irradiations (Figure 5a), where final exposure levels of 40, 60 and 80 J/cm^2^ were achieved. Subsequently, mtDNA was isolated, and qPCR analysis was performed to quantify levels of the CD. Further, the CD levels were determined separately in both the epidermal and dermal layers, i.e., in keratinocytes and fibroblasts, respectively.

Upon dissociation of the epidermal and dermal layer in the HSE, an increase in the CD upon UVA exposure was observed in both skin layers (Figure 5b). The highest CD levels (70%) were observed for keratinocytes exposed to the lowest UVA dose (40 J/ cm^2^), whereas the relative CD levels decreased with higher UVA doses (60 and 80 J/ cm^2^). For fibroblasts, the highest relative CD levels were reached at the dose of 60 J/cm^2^ and 80 J/cm^2^ (Figure 5b). To assess whether these exposure levels affected tissue integrity, hematoxylin-eosin staining was performed. The structure of the HSE was not significantly perturbed by 40 or 60 J/ cm^2^ UVA, while separation of the epidermis from the dermis started to be observed at 80 J/ cm^2^ (Figure 5c). Consistent with the findings in the fibroblast monolayer (Figure 2c), dot blot analysis using an anti-8-oxo-dG antibody showed an increase in 8-oxo-dG in both layers of the HSE (Figure 5d and Figure S8). Overall, UVA-induced CD formation was observed in keratinocytes and fibroblasts along with an increase in mtDNA base oxidation, although the extent of CD levels and the kinetics were distinct between the two skin layers.

To confirm the anticipated protective effects of antioxidants on the formation of the CD (Figure 3), we exposed HSE for two days to 2 × 10 J/ cm^2^ UVA, reaching a maximum accumulated irradiation exposure of 40 J/ cm^2^ UVA, either in the presence or absence of CoQ10 (Figure 5e). Whereas UVA exposure increased CD in keratinocytes to a level of 70%, CoQ10 reduced it to 45%. In fibroblasts, CD levels following UVA exposure were 55% and were also reduced (28%) by the presence of CoQ10 (Figure 5f). In conclusion, we demonstrated that UVA-induced mtDNA CD can be measured in keratinocytes and fibroblasts in a 3D skin model and that these effects can be mitigated by supplementation with antioxidants. These findings support further investigation of mtDNA CD as a potential biomarker for UVA-induced mitochondrial damage while highlighting the potential of the HSE model for in vitro testing.

## 4. Discussion

Our findings characterize the impact of UV radiation on human skin cells and highlight mtDNA base damage in the context of UV-induced skin alterations and their mitigation. We delineated the relationship between increasing levels of UVA radiation, ROS production, mtDNA base oxidation and the accumulation of the mtDNA CD in human skin fibroblasts and investigated potential similarities and contrasts with responses to UVB exposure. UVA exposure led to the upregulation of genes involved in mtDNA degradation, the downregulation of repair-related genes, and a reduction in mtDNA-encoded gene expression. Consistent with initiation by UVA-induced ROS formation, we demonstrated that CD levels were reduced when cells were conditioned with common antioxidants GSH and co-enzyme Q10. Finally, using a 3D skin model physiologically similar to human skin, we were able to link CD content changes with UVA exposure and counteract this damage with antioxidants, supporting the CD as a potential biomarker to assess skin photoaging.

UV radiation doses used in this study were at the high end of environmentally relevant exposure levels and were established based on initial dose-finding tracking the upregulation of MMP-1 and downregulation of COL1A1, two genes related to collagen degradation and UV-induced skin damage and photoaging [58,59]. The cumulative UVA dose of 100 J/cm^2^ was delivered over five days at 20 J/cm^2^ per day. Peak summer midday UVA irradiance at mid-latitudes reaches approximately 5.4 mW/cm^2^ (19 J/cm^2^ per hour) [60], meaning each daily dose corresponds to roughly one hour of midday summer sun. As UVA penetrates efficiently into the dermis [61] this comparison is directly relevant to fibroblasts in vivo. Wang et al. [5] used the same daily UVA dose of 20 J/cm^2^ on human skin in vivo and demonstrated progressive dermal damage. The UVB model was included primarily to compare mechanisms of UVA- and UVB-induced mtDNA damage; the cumulative dose of 3 J/cm^2^ is consistent with established in vitro photoaging models [62]. We found that CD content increased in a dose-dependent manner, with UVB exhibiting a more pronounced effect as compared to UVA (Figure 1b). The CD content before and after UV treatments was measured by qPCR and expressed as a relative percentage over total mtDNA content. By measuring citrate synthase activity (Figure 1d), a widely used assay for mitochondrial mass, we found that mitochondria mass, and therefore total mtDNA content, remained unchanged across UV doses. The highest UVA CD levels were observed at 100 J/cm^2^, where cell viability was also reduced to about one third, raising the question of whether the selective survival of cells already harboring CD could contribute to the observed increase. However, cytotoxicity and CD levels lack a consistent correlative relationship throughout the UV models, which argues against selective survival as the sole explanation for CD accumulation. For example, 1 J/cm^2^ UVB increased CD levels to over 60%, with little impact on viability (above 80%), whereas 60 J/cm^2^ UVA yielded only 5% CD yet reduced viability by half, arguing against selective survival as a primary driver. Furthermore, antioxidant exposure reduced CD levels associated with UV irradiation (Figure 3), implicating ROS-mediated de novo CD formation. Together, these observations suggest that selective survival is not the primary driver of the observed CD accumulation, although at the higher doses where cell viability is substantially reduced, it cannot be fully excluded. Taken together, these results suggest that, despite the observed significant decrease in cell viability (Figure 1c), mtDNA depletion alone is unlikely to explain the relative enrichment of CD mtDNA. In treated cells, a notable decline in both OCR and ECAR for the two UV exposures was observed (Figure 1e,f), evaluated at both basal and maximal ETC activity levels, with OCR decline being consistent with previous observations for UVA-exposed fibroblasts [63]. These data contribute to the current view of mitochondrial dysfunction as part of the global stress response to UV and as a hallmark of aging [27] and photoaging in particular [64].

We characterized dynamic aspects of UV-induced ROS, base damage and mtDNA CD formation. Cellular ROS increased with a corresponding accumulation of 8-oxo-dG in mtDNA (Figure S2a and Figure 2); however, ROS levels decreased between 80 and 100 J/cm^2^ UVA, whereas the CD content increased in a dose-dependent manner. This deviation in relative dose–response relationships may reflect reduced mitochondrial metabolic activity at higher UVA exposure levels, increased antioxidant enzyme activity and cellular ROS buffering capacity [65], or initiation of apoptosis in cells harboring higher levels of ROS [66]. Zhivagui et al. also reported that fibroblasts exposed to UVA had elevated levels of 8-oxo-dG and linked this observation with the occurrence of ROS-associated mutational signatures (SBS18/36) as skin cancer driver mutations [67]. In contrast to UVA-induced effects, UVB did not measurably increase cellular ROS, and accordingly, there was the expected high accumulation of CPDs rather than 8-oxo-dG (Figure S2b) [68]. CPD levels persisted for several days, and their gradual reduction mirrored the slower decline of UVB-induced CD (and Figure 2b). What seems to be consistent for both UVA and UVB is that irradiation increased mtDNA base damage (8-oxo-dG for UVA and CPD for UVB) along with increasing levels of the CD and that the kinetic profiles of the decrease of the CD and mtDNA base damage post-exposure are mirrored. The distinct kinetic profiles of UVA- and UVB-induced CD (Figure 2a) may reflect a combination of factors, including differences in the repair capacity for 8-oxo-dG and CPDs, selective removal of damaged mtDNA, or dilution through mtDNA resynthesis. Notably, our RNA-Seq data indicates that resynthesis-driven dilution may also contribute to the observed kinetics (Figure 4b). Finally, since lesion-induced stalling of mtDNA replication can initiate CD formation, we confirmed polymerase γ pausing at 8-oxo-dG (Figure S4), consistent with previous reports [56]. Based on these findings, we propose two distinct initiation processes leading to the UV-dependent formation of the CD in human skin fibroblasts: (i) stalling of polymerase Ɣ at 8-oxo-dG or CPDs [69], allowing the 13bp repeats to mis-hybridize and lead to loop formation and subsequent deletion formation either through replication slippage (23,24) or (ii) copy choice recombination (29).

Given the link between UVA-dependent increases in ROS and CD accumulation, we tested the hypothesis that antioxidants could mitigate this process (Figure 3). Indeed, when cells preconditioned with common antioxidants GSH and CoQ10 were exposed to UVA, CD levels were reduced, consistent with the causal role of ROS in CD formation. Interestingly, we also observed that high concentrations of GSH increase CD levels in the absence of UVA exposure, consistent with previous findings by Fang Ji et al. [70], who reported higher CD formation with GSH above 200 µM. While this effect was attributed previously to GSH acting as an electron donor and thereby promoting ROS generation [70], our data support that GSH lowers cellular ROS relative to baseline (Figure S5). This suggests that the increase in CD observed in unexposed, GSH-treated cells may arise from reductive stress rather than oxidative stress, consistent with findings that glutathione-dependent reductive stress triggers mitochondrial oxidation [71]. While DCF measurements confirmed that GSH (500 µM) reduced ROS below baseline levels (Figure S5a), consistent with a highly reduced cellular environment, this was not confirmed by direct redox status measurements such as NAD+/NADH or GSH/GSSG ratios. Nonetheless, the data cautions that excessive antioxidant supplementation may paradoxically impair mitochondrial integrity, underscoring the importance of dose optimization in potential application contexts. Indeed, reductive stress is known to impair mitochondrial function, which may trigger CD formation independently of ROS, further supporting the model that mitochondrial CD reflects mitochondrial quality and metabolic state [71].

KSS is a rare disease characterized by mitochondrial dysfunction caused by mitochondrial deletions, including the CD [72]. Using a hybrid cell line derived from a KSS donor, we further examined whether the relationship between ROS and CD formation observed in UVA-irradiated fibroblasts is relevant in this pathological context (Figure 3). Consistent with this idea, we observed substantially higher basal ROS levels in KSS cells compared to BJ-5ta normal fibroblasts, in agreement with previous observations by Majora et al. [73]. KSS cells also maintained high steady-state CD levels (50% heteroplasmy), providing a suitable model to assess whether reducing ROS could modulate CD content. Short-term co-incubation of KSS cells with the antioxidants GSH or CoQ10 for 8 h markedly lowered ROS levels, which was accompanied by a substantial reduction in CD abundance. Importantly, the KSS-associated CD is germline in origin and not induced by oxidative stress. The reduction in CD levels observed here therefore reflects a shift in the maintenance of the existing heteroplasmy. Maintenance of mtDNA deletions has been linked to impaired expression of nuclear-encoded respiratory chain factors [74], suggesting that interventions affecting mitochondrial function, such as reducing oxidative stress, can shift the balance between deleted and wild-type mtDNA. The CD is measured as a ratio relative to total mtDNA, meaning that this rapid shift could reflect selective mitophagy of CD-carrying mitochondria, preferential replication of wild-type mtDNA, or both. Antioxidant treatment may relieve ROS-mediated suppression of PINK1/Parkin-dependent mitophagy, enabling clearance of mitochondria enriched in deleted genomes [75]. Alternatively, reducing oxidative stress may shift replication competition in favor of wild-type molecules [76]. Importantly, mtDNA replication is independent of the cell cycle, and synthesis of individual molecules occurs within 1–2 h [77], providing sufficient turnover within the 8 h exposure window to shift heteroplasmy balance.

Although the mechanism underlying the long-term persistence of high CD levels in KSS remains unclear, our findings suggest that elevated basal ROS contributes to maintaining these pathological CD levels. The mechanism for CD formation appears to be similar to UVA-induced CD formation, as CD formation can be reduced by antioxidant supplementation. This is consistent with a potential therapeutic role of antioxidant supplements for KSS patients [78]. Recent work using engineered mtDNA deletions showed that mitochondrial dysfunction arises only once deletion heteroplasmy exceeds a critical threshold, with pronounced defects above 75% [79]. This supports the idea that mtDNA deletions, possibly including the CD, have burden-dependent functional consequences. Although our UVA-induced CD levels remain below such thresholds, these findings highlight those sustained increases in deletion load, whether from oxidative stress or disease, can impair mitochondrial function.

A key finding from the novel transcriptomic analysis presented here concerning gene regulation in UV-induced CD formation is that genes associated with mtDNA replication (*TFAM*, *POLG*, *POLG2*, *SSBP1*, *TWNK*, *POLRMT*, *TEFM*, *TFB2M*, *RNASEH1*) and mtDNA degradation (*PARP1*, *MGME1*, *POLG2*, *TWNK*, *EXOG*, *FEN1*) are upregulated, while base excision repair (*OGG1*, *MUTYH*, *UNG*, *MPG*, *NEIL1*, *NEIL2*, *NTHL1*, *APEX1*, *LIG3*, *PNKP*, *ERCC6*, *ERCC8*, *RAD23A*, *ERCC2*), mismatch repair (*MUTYH*, *UNG*), and double-strand break repair (*MRE11*, *BRCA1*, *DNA2*, *XRCC6*, *XRCC4*, *RAD51*) genes are downregulated (Figure 4). This observation suggests that, upon UVA irradiation, cells prioritize mtDNA turnover over repair. The upregulation of mtDNA replication genes could be an indication of a compensatory mechanism to replace damaged mtDNA, as previously proposed in studies regarding oxidative stress responses [80]. The observed downregulation of DNA repair-associated genes could be an adaptive response to avoid an overload of repair intermediates, which carry the inherent risks of increasing genome instability and indirectly disrupting genome function or causing mutations. We further observed a downregulation of all 13 protein-coding mtDNA genes upon UVA irradiation, including the mitochondrial ribosomal RNA subunits (MT-RNR1 and MT-RNR2), which are essential for mitochondrial protein synthesis. The reduced expression of mitochondrial-encoded genes can be linked with compromised oxidative phosphorylation and enhanced ROS production, leading to a feedback loop of oxidative damage and impaired mitochondrial function. Our comparative analysis with UVB exposure revealed that while mtDNA-encoded genes had similar patterns of downregulation, nuclear-encoded genes involved in mtDNA maintenance had distinct expression profiles, depending on the type of UV exposure.

In this study, we measured UVA-induced CD formation and mtDNA base damage in a physiologically relevant 3D skin model (Figure 5). Notably, we demonstrated that supplementing HSE with CoQ10 effectively mitigated the adverse effects of UVA-induced CD formation, highlighting the potential of antioxidants as protective interventions against UV-induced damage and possibly photoaging. Furthermore, our data indicate the potential of HSE as an in vitro model for testing photoaging interventions, including antioxidant therapies. The observation that UVA-induced CD in keratinocyte decreases with increasing UVA exposure, together with the upregulation of mtDNA replication genes upon UVA irradiation (Figure 4a), suggests there is a protective mechanism by which cells with high CD levels are replaced by active proliferation in keratinocytes. Thus, the data in this study support that the UVA-specific initiating mechanism for elevated CD levels is by the promotion of ROS, supporting a molecular basis for how antioxidant interventions can mitigate UV-induced skin damage. Moreover, the 3D HSE model combined with CD monitoring technology establishes a basis for characterizing molecular and cellular responses to targeted interventions to prevent photoaging.

## Data Availability

RNA-Seq raw data files and processed files can be found in the NCBI Gene Expression Omnibus (GEO) (accession number GSE302943). All other data underlying this article will be shared at reasonable request to the corresponding author.

## Supplementary Materials

The following supporting information can be downloaded at: https://www.mdpi.com/article/10.3390/life16040577/s1, Table S1: Primers used in this study; Figure S1: UVA-induced gene expression changes in UV responsive genes; Figure S2: Uncropped dot blots and densitometric quantification; Figure S3: Uncropped dot blots and densitometric quantification; Figure S4: Primer extension assay; Figure S5: ROS measurement with antioxidants Bj5tA and KSS cell lines; Figure S6: UVA RNA-Seq analysis; Figure S7: UVB-induced gene expression changes; Figure S8:Uncropped dot blots and densitometric quantification of 8-oxo-dG in human skin equivalents (HSE).
