# Supplementary material for: UVA Irradiation Promotes ROS-Mediated Formation of the Common Deletion in Mitochondrial DNA
Source: Life (Basel). 2026 Apr 1;16(4):577. doi: 10.3390/life16040577 (PMC13117788; doi:10.3390/life16040577)
Supplement: Supplementary file 1 [file life-16-00577-s001.zip › life-4156019-supplementary.pdf]

Supplementary

Table S1: Primers used in this study

**Primers for qPCR-based analysis of mtDNA species**			
**Target**	**Forward sequence (5'->3')**	**Reverse sequence (5'->3')**	**Reference**
mtDNA total	TAGCCCTAAACCTCAACAGT	TGCGCTTACTTTGTAGCCTTCAT	[1]
mtDNA CD	TTCCTCATCACCCAACTAAAAA	TTCGATGATGTGGTCTTTGG	[1]
mtDNA undeleted	TCGATGATGTGGTCTTTGGA	CATCTGTACCCACGCCTTCT	[2]
ACTB genomic	TCACCCACACTGTGCCCATCTACGA	CAGCGGAACCGCTCATTGCCAATGG	[2]
**Primers for qPCR-based analysis of gene level expression in cDNA**			
ACTB genomic	TCACCCACACTGTGCCCATCTACGA	CAGCGGAACCGCTCATTGCCAATGG	[2]
APEX1	GAGGAGCATGATCAGGAAGG	GCTGTTACCAGCACAAACGA	[2]
BRCA1	AGAGTCCAGCTGCTGCTCAT	CCCTGCTCACACTTTCTTCC	[2]
DNA2	AGCTCTTGGCATGAGTGAAAG	GCACGGTTAACTGTACAACAGC	[2]
ERCC2	TTCTCTGGGCTCGACGAC	AGTCGTACGGGAAGTAGACCAG	[2]
ERCC6	GGAGCAGAGGTGAAAATTGAA	CTCCTGGACAGGCATGAG	[2]
ERCC8	CAAGTCACAGACAAGAAATATTAGCAG	AGCACTTGCTGTTGCCAAG	[2]
EXOG	GGCTCCAGCAGGAAATAAC	CAAAATCCTGAGGCACAATG	[2]
FEN1	TCGAACTTGCTATGTAATTTGTGTC	AACTCAGCTGATTGCCAGGT	[2]
GAPDH	ACGGATTTGGTCGTATTGGG	TGATTTTGGAGGGATCTCG	[2]
HUMANIN	CGAGGGTTCAGCTGTCTCTT	GGCAGGTCAATTTCACTGGT	[2]
LIG3	CAACACGAAGACCCAGATCA	GGTACACATCACCGTGGAAA	[2]
MGME1	GACTGAAAAGCCCCAAAGTCT	GCGTGTCCTCCTTTCAGGTA	[2]
MPG	TTTACGGCATGTACTTCTGCAT	ATGGTCTCCAGACCTTCCAG	[2]
MRE11	ACAACCTGGAAGCTCAGTGG	TTAATACGCAGCAAACCAACA	[2]
mtRNA UTR	CTTTGATTCCTGCCTCATCC	TGATGTCTGTGTGGAAAGTGG	[2]
MT-ATP6	CCACAATCCTAGGCCTACCC	GGGATCAATAGAGGGGGAAA	[2]
MT-ATP8	GCCTACTCATTCAACCAATAGC	TCAGTAGAATTAGAATTGTGAAGATGA	[2]
MT-CO1	ATCCTACCAGGCTTCGGAAT	CGGAGGTGAAATATGCTCGT	[2]
MT-CO2	CCATCCCTACGCATCCTTTA	GGTCGCCTGGTTCTAGGAAT	[2]
MT-CO3	CCCGCTAAATCCCCTAGAAG	ATGGTGAAGGGAGACTCGAA	[2]
MT-CYB	TATCCGCCATCCCATACATT	GGTGATTCCTAGGGGGTTGT	[2]
MT-ND1	TGAAGTCACCCTAGCCATCA	GGTTCGGTTGGTCTCTGCTA	[2]
MT-ND2	AAGCAACCGCATCCATAATC	TCAGAAGTGAAAGGGGGCTA	[2]
MT-ND3	CCACAACTCAACGGCTACAT	TTGTAGGGCTCATGGTAGGG	[2]
MT-ND4	CTCGCTAACCTCGCCTTACC	AGTGAGCCCCATTGTGTTGT	[2]
MT-ND4L	TCGCTCACACCTCATATCCT	GCCATATGTGTTGGAGATTGA	[2]
MT-ND5	CGCTTCCCCACCCTTACT	GCGAGGGCTGTGAGTTTTAG	[2]
MT-ND6	TCTGAATTTTGGGGGAGGTT	CCACAGCACCAATCCTACCT	[2]
MT-RNR1	AAACGCTTAGCCTAGCCACA	CTTTACGCCGGCTTCTATTG	[2]
MT-RNR2	ACTTTGCAAGGAGAGCCAAA	TGGACAACCAGCTATCACCA	[2]
MUTYH	GTGTGTATCAGGGCCAACAG	GACTGCACGGAGAGGACAC	[2]
OGG1	TGTCACCTACCATGGCTTCC	AGGCCCAGCTTCCTGAGA	[2]
NEIL1	AGCTGCGCCTGATACTGAG	GCTGAAAAGAGCCGGACAT	[2]
NEIL2	GGGCAGCAGTAAGAAGCTACA	TGCAGGACCAACCTCACC	[2]
NTHL1	AGGTGCTGCTGTCACTGATG	TCTGCAGGATGCTGTCCA	[2]
PARP1	GGGATGACCAGCAGAAAGTC	CTGCCTTGCTACCAATTCC	[2]

PNKP	GACAGCATCTTTGTGGGAGAC	AAGGTTGAGGGCAAACAGG	[2]
POLG	CAGGTACCACCCTGGAGTC	CCCTGTTCGAGACAGTGCTT	[2]
POLG2	CACGAACTTTTACACATGTATCC	CAGAGAGAACACAAGGAACC	[2]
POLRMT	CATGTACAACGCCGTGATG	GGCATCCTTCACCATGAATAA	[2]
RAD23A	ATGCGGCAGGTGATTCAG	AGGGCCTTCAACTGTAAAAGC	[2]
RAD51	GGGAATTAGTGAAGCCAAAGC	TGGTGAAACCCATTGGAACT	[2]
RNASEH1	GGCCTTTGTCAGGAAATCTG	CTTTCGCCTCCGATTCTTGT	[2]
SSBP1	CATGAGTCCGAAACAACTACCA	ACAGGGTCCTGACCCACTC	[2]
TEFM	GAGAAAGCTCCTCAAACCAG	CAATTCTTCGAGTACCAAAAACG	[2]
TFAM	TGCAACTTCTGTGGAAGCAT	GAATCAGGAAGTTCCCTCCA	[2]
TFB2M	AAATTTGGACGAATAGAAGTAAATATG	AGTCTGGATTTCCGGGATCT	[2]
TOP1MT	CACAACAAAGGAGGTTTTCC	CCAGGCTCTTGATGACTTCC	[2]
TOP3A	GCCCAAGAGCAAGTGGCG	CCTCATGGTTTCTTTAGCATTT	[2]
TWNK	GGACCTGCCCCTCTATTTC	CATAGACGTAGACTGCATGTTGC	[2]
UNG	GCTGAGTGCCGAGCAGTT	TGCTTCTTCCAGCTCTCTCC	[2]
XRCC4	GAGGTAGGATCCGGAAGTGG	GAAACAAGGTGGATTCTGCTTA	[2]
XRCC6	GCCTTGTCCTCAGCCAGTTA	CCTCGACTTATGTCGGGTAGA	[2]
YBX1	CAATGTAAGGAACGGATATGG	GGTGTACAAATACATCTTCCTTGG	[2]

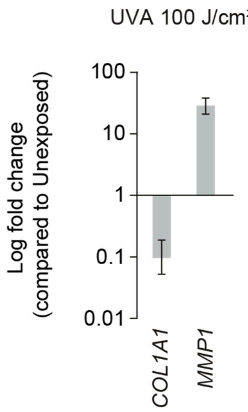

Figure S1 | UVA-induced gene expression changes in UV responsive genes. qPCR-based analysis of expression of *COL1A1 and MMP1* upon treatment of Bj5-ta cells with 100 J/cm^2 as shown Figure 1a. n = 3.

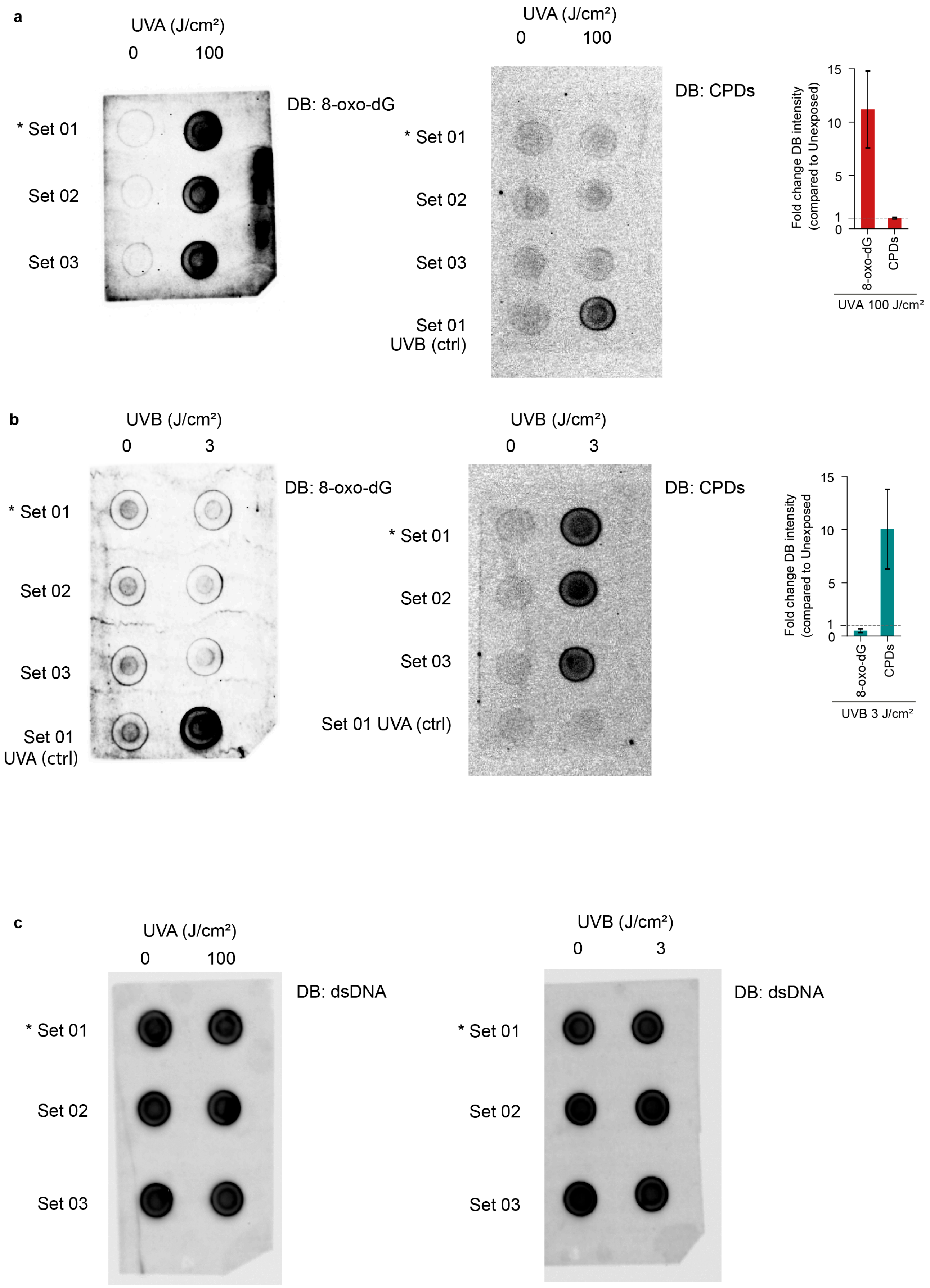
a
UVA (J/cm²)
0
100
DB: 8-oxo-dG
* Set 01
Set 02
Set 03
UVA (J/cm²)
0
100
DB: CPDs
* Set 01
Set 02
Set 03
Set 01
UVB (ctrl)
Fold change DB intensity
(compared to Unexposed)
15
10
5
1
0
8-oxo-dG
CPDs
UVA 100 J/cm²
b
UVB (J/cm²)
0
3
DB: 8-oxo-dG
* Set 01
Set 02
Set 03
Set 01
UVA (ctrl)
UVB (J/cm²)
0
3
DB: CPDs
* Set 01
Set 02
Set 03
Set 01 UVA (ctrl)
Fold change DB intensity
(compared to Unexposed
15
10
5
1
0
8-oxo-dG
CPDs
UVB 3 J/cm²
c
UVA (J/cm²)
0
100
DB: dsDNA
* Set 01
Set 02
Set 03
UVB (J/cm²)
0
3
DB: dsDNA
* Set 01
Set 02
Set 03

Figure S2 | Uncropped dot blots and densitometric quantification**.** Dot blot analysis was performed on mtDNA extracted from BJ-5ta cells irradiated with (a) UVA (100 J/cm^2) or (b) UVB (3 J/cm^2). Hybridizations were carried out using anti-8-oxo-dG (left) and anti-cyclobutane pyrimidine dimer (CPD, middle) antibodies. Bar graphs (right) show densitometric quantification of dot blot intensity as fold change relative to the unexposed condition. (c) Anti-double stranded DNA (dsDNA) dot blots used as loading controls for UVA (left) and UVB (right). Set 01, Set 02, and Set 03 represent three independent biological replicates.

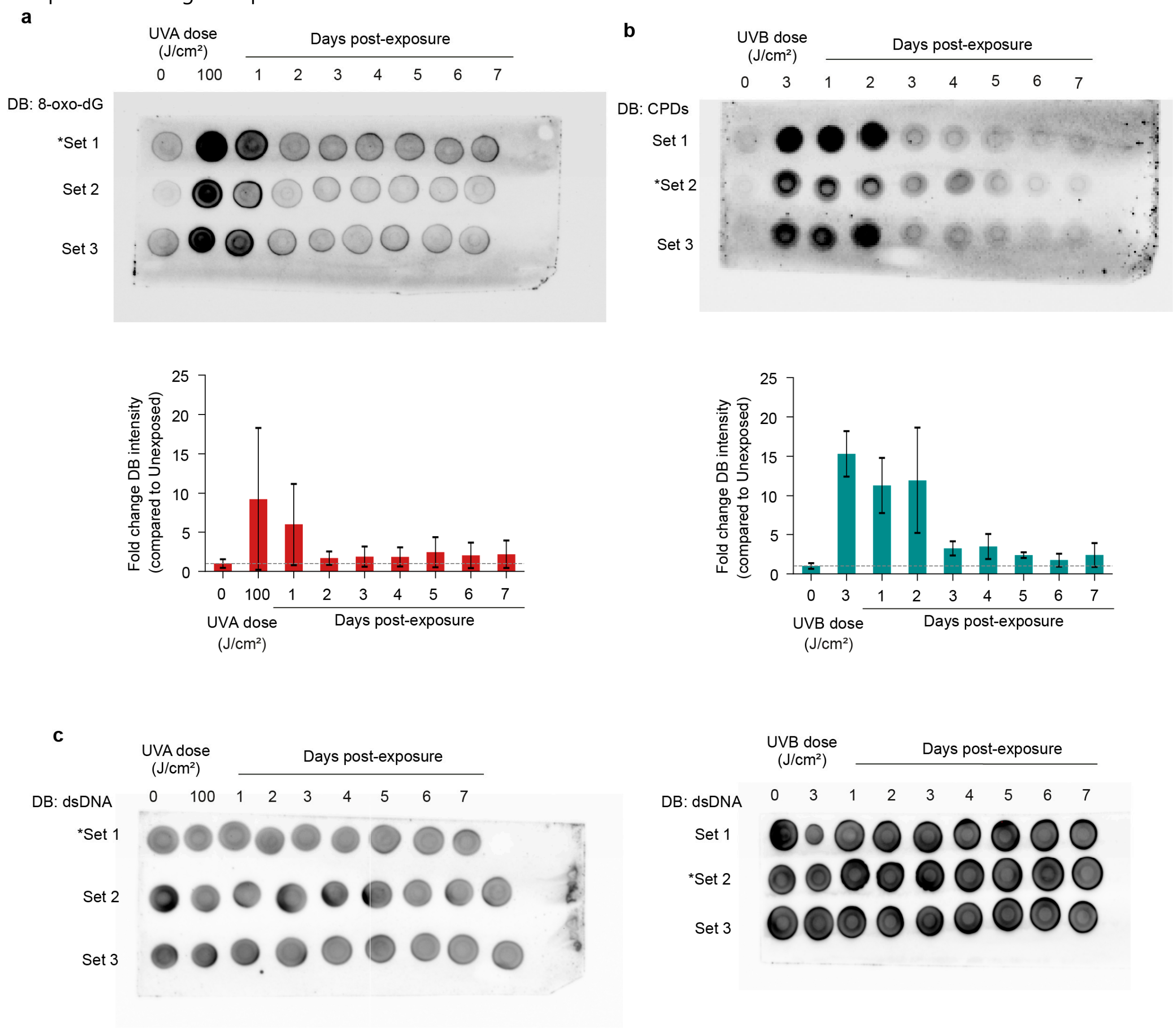

Figure S3 | Uncropped dot blots and densitometric quantification. DBs were performed on mtDNA extracted from BJ-5ta cells irradiated with the indicated doses of **(a)** UVA (100 J/cm^2) or **(b)** UVB (3 J/cm^2) and collected every 24 h for seven days after exposure in absence of further stimuli. Hybridizations were carried out using **(a)** anti-8-oxo-dG, **(b)** anti-cyclobutane pyrimidine dimer (CPD), and **(c)** anti-double stranded DNA (dsDNA) antibodies as a loading control. Bar graphs show densitometric quantification of dot blot intensity expressed as fold change relative to the unexposed condition. Set 1, Set 2, and Set 3 represent three independent biological replicates. Error bars represent standard deviation (n=3).

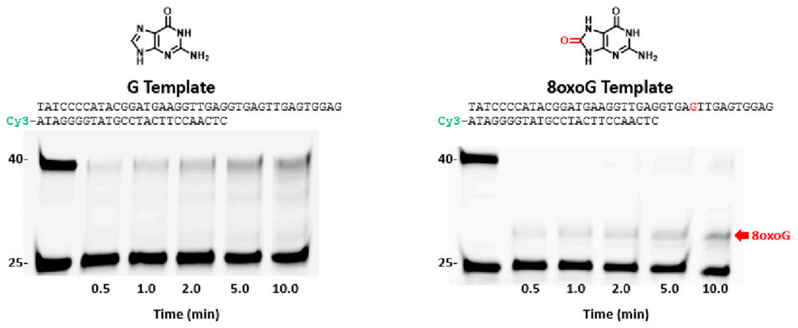

Figure S4 | Primer extension assay. Primer extension assays with mitochondrial DNA Pol γ replication past 40mer templates containing either an unmodified G Template (left) or a modified 8-oxo-dG template (right). Templates were pre-annealed opposite 25mer primers containing a 5′ Cy3 dye.

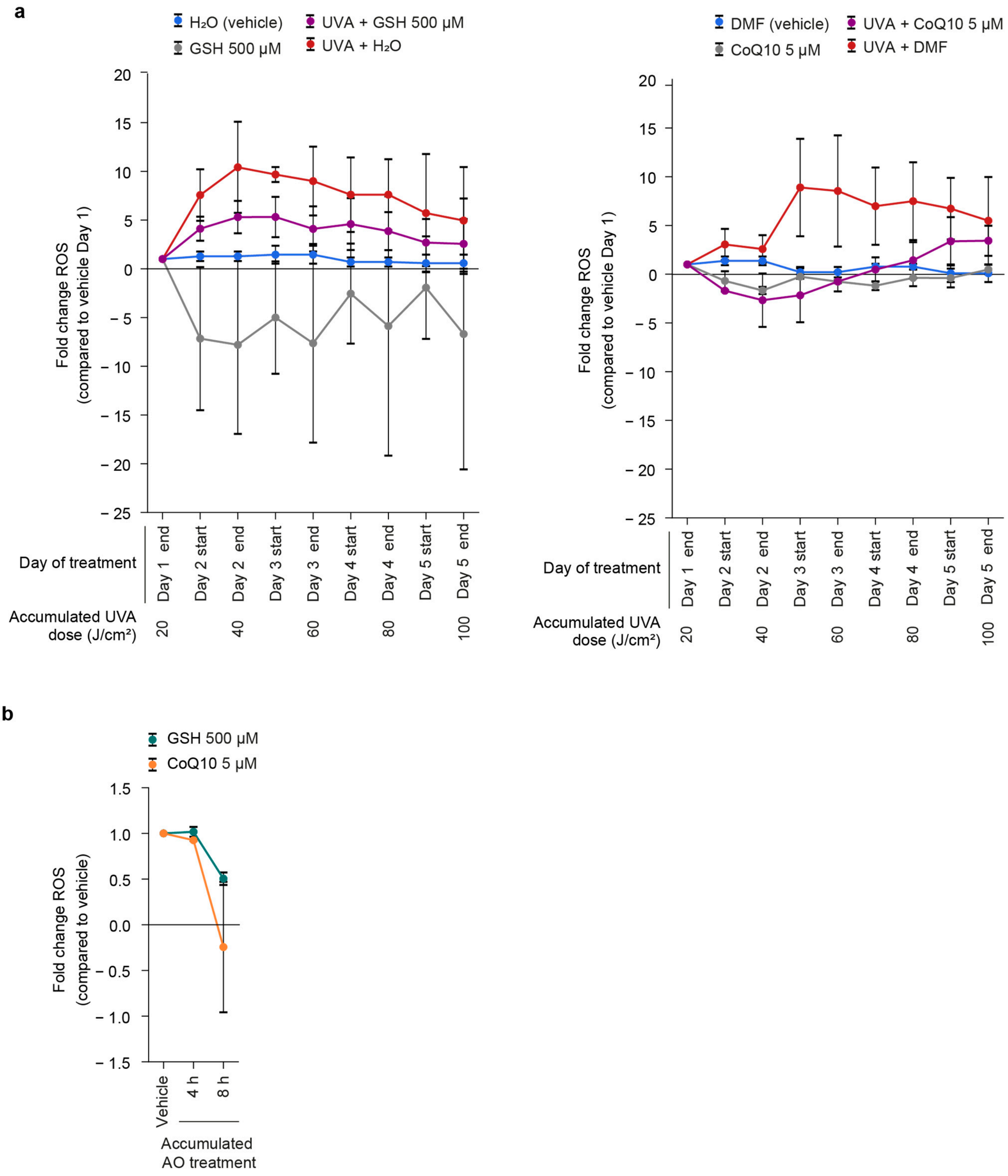

Figure S5 | ROS measurement with antioxidants Bj5tA and KSS cell lines

(**a**) Cellular ROS content in BJ-5ta under-going the co-treatment with reduced glutathione (GSH, left), Coenzyme Q10 (CoQ10, right) and/or the corresponding vehicles H2O or Dimethylformamide (DMF). Cells were collected at the start and at the end of the UV exposure procedure and the ROS content was quantified using the DCF assay. Data are expressed as fold change to cells exposed to vehicle at the end of the first day. **(b)** ROS content in KSS cells treated with vehicle or with GSH or CoQ10 for 4 h per day over 2 days. n = 3 biological replicated for panel (a.b). Error bars represent standard deviations.

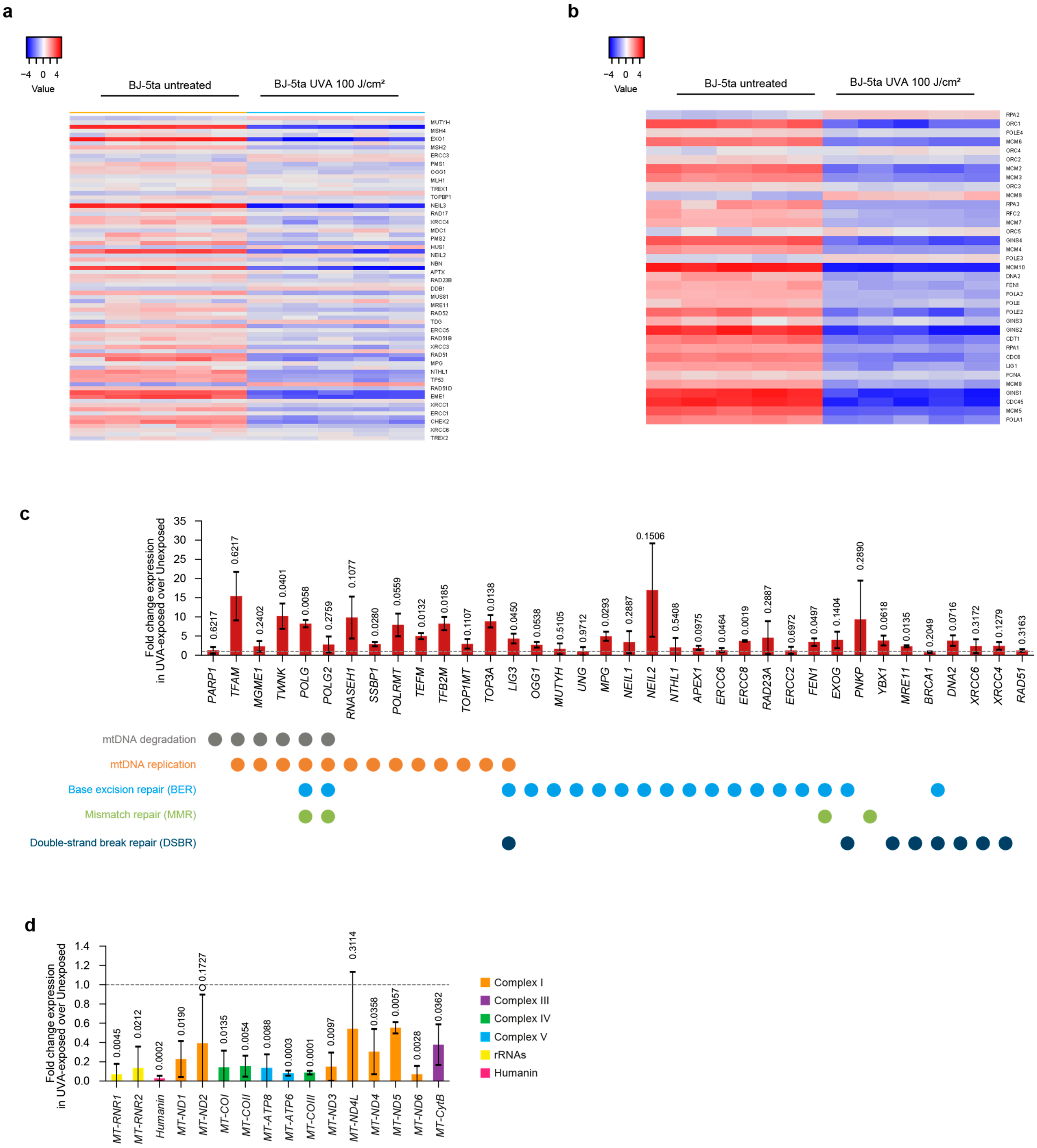

Figure S6 | UVA RNA-Seq analysis. UV-irradiation (100 J/cm^2) induced gene expression changes in nuclear genes encoded for **(a)** DNA repair and **(b)** for DNA replication. 5 **(c)** qPCR assays for expression of mtDNA maintenance genes upon UVA-exposed cells. n = 3 biological replicates were used for each graph. Statistical analysis was performed with a paired t-test comparing UV-exposed samples to matched unexposed control. **(d)** qPCR analysis for the expression of mtDNA-encoded genes in UVA-exposed cells, expressed as fold change over unexposed cells (grey dotted line). Color codes indicate the ETC complexes to which the encoded proteins belong to, rRNAs and the Humanin micropeptide. n = 3 biological replicates were used for each graph. Statistical analysis was performed with a paired t-test comparing UV-exposed samples to matched unexposed control and p-values are indicated above each bar.

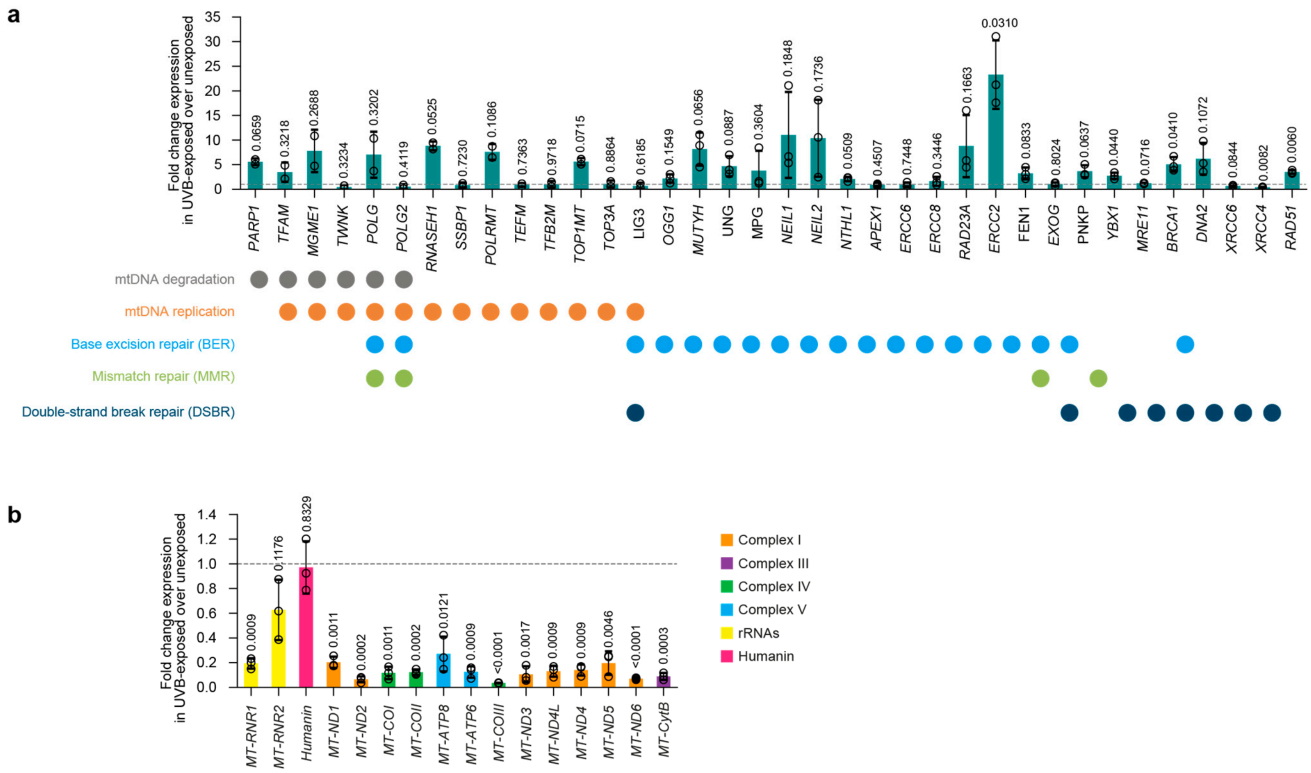

Figure S7 | UVB-induced gene expression changes. qPCR analysis of **(a)** nuclear-encoded mtDNA maintenance genes and **(b)** mtDNA-encoded genes in UVB-exposed cells. Data are expressed as fold change relative to unexposed cells (indicated by the grey dotted line). Statistical significance was determined using a paired t-test, comparing UVB-exposed samples to matched unexposed controls and p-values are indicated above each bar.

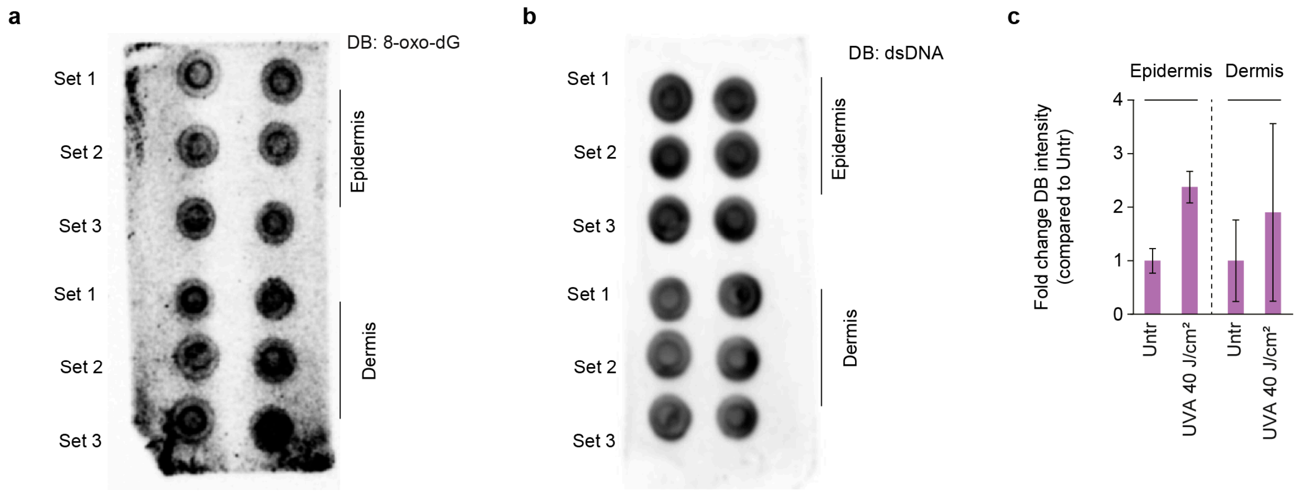

Figure S8 | Uncropped dot blots and densitometric quantification of 8-oxo-dG in human skin equivalents (HSE). Dot blot analysis was performed on mtDNA extracted from the epidermis and dermis of HSE, untreated or exposed to 40 J/cm^2 UVA. Hybridizations were carried out using (a) anti-8-oxo-dG and (b) anti-double stranded DNA (dsDNA) antibodies as a loading control. (c) Densitometric quantification of dot blot intensity expressed as fold change relative to the untreated condition for epidermis (left) and dermis (right). Set 1, Set 2, and Set 3 represent three independent biological replicates. Error bars represent standard deviation (n=3).

1. Phillips AF, Millet AR, Tigano M, Dubois SM, Crimmins H, Babin L, et al. Single-Molecule Analysis of mtDNA Replication Uncovers the Basis of the Common Deletion. Mol Cell. 2017;65(3):527-38.e6.
2. Fontana GA, MacArthur MR, Rotankova N, Di Filippo M, Beer HD, Gahlon HL. The mitochondrial DNA common deletion as a potential biomarker of cancer-associated fibroblasts from skin basal and squamous cell carcinomas. Sci Rep. 2024;14(1):553.
